# A New Approach of Detecting *ALK* Fusion Oncogenes by RNA Sequencing Exon Coverage Analysis

**DOI:** 10.3390/cancers16223851

**Published:** 2024-11-16

**Authors:** Galina Zakharova, Maria Suntsova, Elizaveta Rabushko, Tharaa Mohammad, Alexey Drobyshev, Alexander Seryakov, Elena Poddubskaya, Alexey Moisseev, Anastasia Smirnova, Maxim Sorokin, Victor Tkachev, Alexander Simonov, Egor Guguchkin, Evgeny Karpulevich, Anton Buzdin

**Affiliations:** 1 Institute for Personalized Oncology, World-Class Research Center “Digital Biodesign and Personalized Healthcare”, I.M. Sechenov First Moscow State Medical University, 119991 Moscow, Russia; galina.s.zakharova@gmail.com (G.Z.); suntsova_m_v@staff.sechenov.ru (M.S.); elnrabush@gmail.com (E.R.); drobyshev_a_l@staff.sechenov.ru (A.D.); podd-elena@ya.ru (E.P.); moiseev_a_a@staff.sechenov.ru (A.M.); liliumanstist@gmail.com (A.S.); sorokin@oncobox.com (M.S.); simonov@oncobox.com (A.S.); 2 Endocrinology Research Center, 117292 Moscow, Russia; tharaamohammad1996@gmail.com; 3 Medical Holding SM-Clinic, 105120 Moscow, Russia; alseryakov@yandex.ru; 4 Clinical Center Vitamed, 121309 Moscow, Russia; 5 Oncobox LLC, 119991 Moscow, Russia; tkachev@oncobox.com; 6 Institute for System Programming of RAS, 109004 Moscow, Russia; guguchkin@ispras.ru (E.G.); karpulevich@ispras.ru (E.K.); 7 Shemyakin-Ovchinnikov Institute of Bioorganic Chemistry, 117997 Moscow, Russia; 8 PathoBiology Group, European Organization for Research and Treatment of Cancer (EORTC), 1200 Brussels, Belgium

**Keywords:** fusion oncogene, receptor tyrosine kinase, anaplastic lymphoma kinase (*ALK*), *ALK* rearrangement, RNA sequencing, tumor molecular diagnostics, clinical oncology

## Abstract

Background: In clinical practice, various methods are used to identify *ALK* gene rearrangements in tumor samples, ranging from “classic” techniques, such as IHC, FISH, and RT-qPCR, to more advanced highly multiplexed approaches, such as NanoString technology and NGS panels. Each of these methods has its own advantages and disadvantages, but they share the drawback of detecting only a restricted (although sometimes quite extensive) set of preselected biomarkers. At the same time, whole transcriptome sequencing (WTS, RNAseq) can, in principle, be used to detect gene fusions while simultaneously analyzing an incomparably wide range of tumor characteristics. However, WTS is not widely used in practice due to purely analytical limitations and the high complexity of bioinformatic analysis, which requires considerable expertise. In particular, methods to detect gene fusions in RNAseq data rely on the identification of chimeric reads. However, the typically low number of true fusion reads in RNAseq limits its sensitivity. In a previous study, we observed asymmetry in the RNAseq exon coverage of the 3′ partners of some fusion transcripts. In this study, we conducted a comprehensive evaluation of the accuracy of *ALK* fusion detection through an analysis of differences in the coverage of its tyrosine kinase exons. Methods: A total of 906 human cancer biosamples were subjected to analysis using experimental RNAseq data, with the objective of determining the extent of asymmetry in *ALK* coverage. A total of 50 samples were analyzed, comprising 13 samples with predicted *ALK* fusions and 37 samples without predicted *ALK* fusions. These samples were assessed by targeted sequencing with two NGS panels that were specifically designed to detect fusion transcripts (the TruSight RNA Fusion Panel and the OncoFu Elite panel). Results: *ALK* fusions were confirmed in 11 out of the 13 predicted cases, with an overall accuracy of 96% (sensitivity 100%, specificity 94.9%). Two discordant cases exhibited low *ALK* coverage depth, which could be addressed algorithmically to enhance the accuracy of the results. It was also important to consider read strand specificity due to the presence of antisense transcripts involving parts of *ALK*. In a limited patient sample undergoing ALK-targeted therapy, the algorithm successfully predicted treatment efficacy. Conclusions: RNAseq exon coverage analysis can effectively detect *ALK* rearrangements.

## Simple Summary

Chimeric transcripts frequently function as oncogenic drivers and represent potential targets for tumor-specific therapies. Routine methods for detecting these transcripts include (i) approaches that can detect a limited number of pre-defined targets, such as immunohistochemistry, fluorescence in situ hybridization, and reverse transcription–quantitative polymerase chain reaction, and (ii) approaches that can simultaneously detect multiple fusions, such as NanoString assays and NGS targeted panels. Whole transcriptome sequencing enables the analysis of a wide range of cancer biomarkers, including dysregulated genes, molecular pathways, and both known and novel cancer driver fusion transcripts. However, this method is not commonly employed for fusion discovery due to inadequate coverage at the fusion breakpoint. To address this limitation, we have developed a novel bioinformatics approach that enables the highly accurate prediction of clinically significant *ALK* fusions from RNA sequencing data. This extends the functionality of whole transcriptome NGS and improves its cost-effectiveness.

## 1. Introduction

Chromosomal rearrangements resulting in the formation of transcribed fusion genes are a common source of driver mutations in a wide range of cancer types [1,2]. The identification of functional tyrosine kinase (TK) fusions is of great importance in clinical practice, as TK inhibitors (TKIs) have demonstrated significant efficacy in the treatment of cancers harboring these fusions [3,4,5]. The most frequently observed therapeutic target fusions involve *ALK* (anaplastic lymphoma kinase), *ROS1* (ROS proto-oncogene 1), *RET* (ret proto-oncogene), *NTRK1-3* (neurotrophic receptor tyrosine kinases 1-3), *BRAF* (B-raf proto-oncogene), *EFGR* (epidermal growth factor receptor), and *ABL1* (ABL proto-oncogene 1) genes [6,7].

The *ALK* gene encodes a receptor tyrosine kinase (RTK) that plays a key role in fetal neuronal development [8,9]. In adults, *ALK* is expressed in limited quantities in a restricted number of tissues, including the brain, pituitary gland, and testis, as indicated by the Human Protein Atlas project (https://www.proteinatlas.org/ENSG00000171094-ALK/tissue, accessed on 13 September 2024) [10].

ALK was initially identified as a component of a fusion protein in anaplastic large cell lymphomas, which led to its designation as a specific kinase [11,12]. In pan-cancer studies, approximately 0.2–0.8% of cases were found to have *ALK* rearrangements [13,14]. In anaplastic large cell lymphoma, the frequency of *ALK* fusions is approximately 50–80% [15], with a similar prevalence observed in inflammatory myofibroblastic tumors at around 50–60% [16,17]. In solid tumors, *ALK* rearrangements are most commonly observed in non-small cell lung cancer (NSCLC), occurring in about 3–8% of cases [13,18].

**Figure 1:**
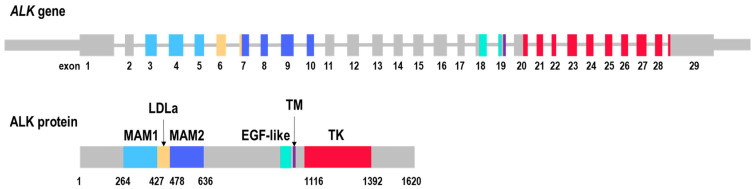
Structure of the wild-type *ALK* gene and the corresponding protein. MAM—methylthioalkymalate synthase-like domain; LDLa—low-density lipoprotein receptor class A; EGF-like—epidermal growth factor-like domain; TM—transmembrane domain; and TK—tyrosine kinase domain.

**Figure 2:**
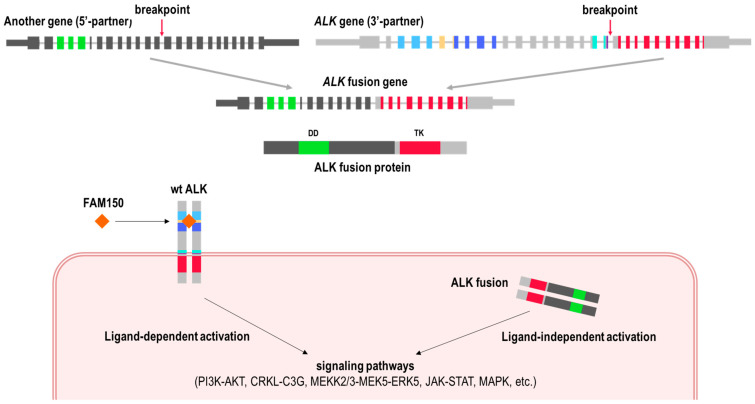
Schematic representation of the formation and functional role of an *ALK* fusion. FAM150—ALK ligand Augmentor α (FAM150A) or Augmentor β (FAM150B); DD—dimerization domain; and TK—tyrosine kinase domain.

The *ALK* gene is located on chromosome 2p23 and consists of 29 exons (Figure 1). The catalytically active tyrosine kinase (TK) domain is encoded by exons 20–29. The formation of oncogenic *ALK* fusions can occur through a number of different mechanisms, as reviewed in detail elsewhere [19]. The *ALK* breakpoint is most frequently located in intron 19 [20,21], resulting in the loss of extracellular and transmembrane domains. The resulting fusion transcripts maintain the TK domain of *ALK* at the 3′-end, while the 5′-partner genes exhibit variability. Among the most frequent 5′-partners are *EML4* (echinoderm microtubule-associated protein-like 4) in NSCLC and *STRN* (striatin), *NPM1* (nucleophosmin), and *TPM3* (tropomyscin 3) in non-NSCLC neoplasms [22,23]. It has been established that nearly all known 5′-partner genes contain oligomerization domains, which result in the constitutive activation of ALK [19]. This results in cellular transformation, uncontrolled growth, lack of normal differentiation, and clonal expansion, as ALK is involved in key proliferative signaling pathways, including Ras/extracellular signal-regulated kinase (ERK), phosphatidylinositol 3 kinase (PI3K)/Akt, and Janus protein tyrosine kinase (JAK)/STAT [24,25,26] (Figure 2).

In clinical practice, a number of methods are commonly employed for the identification of *ALK* fusions, including immunohistochemistry (IHC), immunocytochemistry (ICC), fluorescence in situ hybridization (FISH), real-time polymerase chain reaction with reverse transcription (RT-qPCR), high-throughput nucleic acid hybridization technology (NanoString, Seattle, WA, USA), and DNA- and RNA-based targeted next-generation sequencing (NGS) [7,27,28].

IHC and FISH testing for *ALK* rearrangements frequently yield discrepant results [14,29,30]. This discrepancy is not solely attributable to inherent differences in the sensitivity and specificity of these methods or to the potential influence of human error. Rather, it is a consequence of the fact that the assays in question target biologically distinct features. While FISH is capable of detecting chromosomal rearrangements, it is unable to ascertain whether a functional fusion transcript has been formed. In contrast, IHC is able to detect an elevated expression of the carboxy terminus of ALK, which may result not only from chromosomal translocation but also from overexpression of a wild-type protein or a short transcript variant *ALK*^ATI^ [31].

While some studies indicate that IHC results are more efficacious than FISH in predicting potential responsiveness to tyrosine kinase inhibitor therapy [32,33], this methodology exhibits moderate sensitivity compared to NGS [34]. The issues of abnormal staining and interpretative uncertainties in IHC results underscore the necessity of standardized diagnostic practices, robust internal quality control, and consistent quality monitoring [35], which may prove challenging to implement in all diagnostic laboratories.

Another frequently used method for *ALK* fusion testing is RT-qPCR [14,36,37]. RT-qPCR demonstrates high sensitivity and specificity [36,38]. Compared to the RT-qPCR results, FISH has low sensitivity (about 58%) but excellent specificity (about 99%), and IHC staining has high sensitivity (about 92%) and modest specificity (about 80%) [14]. However, the crucial drawback of RT-qPCR is that it can only capture a small subset of fusion variants, predominantly those that are already well characterized and frequently occurring. At the same time, according to the COSMIC [20] (https://cancer.sanger.ac.uk/cosmic, accessed on 2 July 2024) and FusionGDB 2.0 [21] (https://compbio.uth.edu/FusionGDB2/, accessed on 2 July 2024) databases, more than 25 fusion partner genes for *ALK* have been identified with different breakpoints, further increasing the diversity of *ALK* fusions [39].

The nCounter platform (NanoString, Seattle, WA, USA) employs an amplification-free, high-throughput nucleic acid hybridization technology for the purpose of fusion detection. In contrast to the aforementioned methodologies, NanoString enables multiplexed analysis, allowing for the simultaneous detection of tens or even hundreds of unique fusions [40]. It is a relatively cost-effective solution, offers a rapid turnaround time, and provides straightforward result analysis. The nCounter results show strong agreement with IHC, FISH, and RT-qPCR findings, with concordance rates of 98.5%, 87%, and 85.7%, respectively [41]. However, like RT-qPCR, detection with NanoString assays is limited to pre-defined targets. Additionally, the performance of the NanoString assay varies across tumor types and genes [42] and depends on the probe list and its effectiveness.

Next-generation sequencing (NGS) is increasingly being incorporated into routine practice for the study of tumor biomarkers. Fusions can be detected by both DNA- and RNA-based NGS methods. The latter allows for an assessment of the functionality of the rearrangement, including the presence of the transcript, maintenance of the reading frame, and evaluation of the integrity of the kinase domain. Thus, RNA-based NGS appears to be the preferred choice.

A variety of RNA-based fusion detection panels employ diverse approaches to target enrichment, including (i) highly multiplexed PCR amplification of target regions, as in the Oncomine and Ion AmpliSeq panels (Thermo Fisher Scientific, Waltham, MA, USA); (ii) multiplexed anchored PCR, as in the FusionPlex panel (Archer, San Jose, CA, USA); (iii) single-primer enrichment technology (SPET), as in the ovation fusion panel (NuGEN, San Carlos, CA, USA); and (iv) probe-based hybridization enrichment, as in the TruSight panel (Illumina, San Diego, CA, USA). With the exception of the first method, all of these enrichment techniques allow for the sequencing of all fusions involving target genes, even with unknown fusion partners. NGS panels have demonstrated high sensitivity and specificity for fusion detection [43,44,45]. The European Society for Medical Oncology (ESMO) recommends their use in routine practice for analyzing fusions in NSCLC samples [46]. However, NGS panels also have some drawbacks, including their labor-intensive nature and relatively high cost. The limited number of targets is advantageous in that it increases the sensitivity of detection and simplifies the processing and interpretation of results. However, it is also a disadvantage because it provides only constrained information about the tumor.

In contrast, total RNA sequencing (RNAseq) represents the most comprehensive approach to assessing tumor characteristics, yet it remains the least common. While fusion detection is possible with RNAseq, the number of reads supporting a fusion event is often very low (1–5 junction reads), which presents a challenge in distinguishing true fusions from random DNA fragment chimeras that can arise during library preparation.

In our previous study [47], we observed a distinct asymmetry in exon coverage in RNAseq data from experimental samples with tyrosine kinase gene fusions, as confirmed by RT-qPCR. Specifically, it was observed that in a fused transcript with the TK domain located at the 3′ end, the depth of coverage preceding the breakpoint was significantly less than that observed following it. Given that *ALK* expression is typically low or undetectable in most normal tissues, rearrangement of this gene should lead to a marked increase in the expression of 3′ exons, as has been previously observed for the RTK genes *RET* and *ROS1* [47].

A review of the literature revealed the existence of several analytical assays that use coverage imbalance assessment to detect fusion.

The first set of assays is based on PCR. For example, in [48], a digital PCR method was used for this purpose, with primer pairs to amplify the junctions of the *ALK* exons 2–3, 17–18, and 22–23. Another work [49] describes a method for detecting *ALK* fusions in circulating plasma tumor RNA by measuring the difference in expression of the exon 20/exon 3 portion using qPCR. Patent US11021758B2 describes a method for detecting *ERG* and *TMPRSS2* dysregulation (including fusion formation) in prostate cancer that is based on differences in expression between the 5′ and 3′ regions of a gene using RT-qPCR with probe detection.

The same principle is used in the multiplex RT-qPCR assay Idylla GeneFusion (Biocartis, Mechelen, Belgium), which measures the expression imbalance between the 5′ PCR and 3′ PCR of six kinase genes (*ALK*, *ROS1*, *RET*, *NTRK1-3*). Idylla GeneFusion also targets a panel of 16 *ALK* fusions, 13 *ROS1* fusions, and 7 *RET* fusions.

Among NGS approaches, certain ultrahigh multiplex PCR amplicon-based Ion AmpliSeq and Oncomine (Thermo Fisher Scientific, Waltham, MA, USA) panels offer 5′-partner-agnostic fusion detection for *ALK*, *ROS1*, *RET*, and *NTRK1* using expression imbalance as an additional option to identify fusions [50]. This method is based on either comparing the number of amplicons representing exon–exon junctions of a gene or comparing the levels of amplicons from the 5′ and 3′ regions of a gene.

Limitations of PCR-based approaches include (i) the need to select and validate primers, probes, and PCR conditions for each gene of interest; (ii) the potential for allelic dropout of a primer due to polymorphism, which may result in a false negative; (iii) the fact that when performing reverse transcription and qPCR in a single tube format, as described in patent US11021758B2, amplification of antisense transcripts, if present, also occurs; and (iv) the fact that fusion detection is only possible for a predefined set of target genes.

Another approach to detecting gene fusions based on 5′/3′ imbalance assessment is used in the nCounter platform (NanoString, Seattle, WA, USA). This involves hybridization with multiple fluorescence-labeled probes targeting a 5′ region upstream of the kinase domain exons and a 3′ region either within these exons or further downstream (with eight probes per *ALK*, *RET*, and *ROS1* in the standard assay). In [51], the custom panel for evaluating fusions for 23 sarcoma-related genes (*ALK*, *BCORL1*, *BCOR*, *BRAF*, *CAMTA1*, *CCNB3*, *CREB3L1*, *CREB3L2*, *CSF1*, *FOSB*, *NFATC2*, *NTRK1-3*, *NUTM1*, *PDGFB*, *PHF1*, *PLAG1*, *RET*, *ROS1*, *STAT6*, *TFE3*, and *USP6*) by 3′/5′ exon imbalance is described. As with PCR-based methods, the NanoString-based approach requires prior selection and validation of probes for all target genes.

Unlike these existing techniques, WTS does not limit the choice of targets and eliminates the need to develop assays for each specific gene. In addition, RNAseq provides comprehensive information beyond fusions alone, providing broader insights into the nature of the tumor.

Therefore, we aimed to systematically evaluate the accuracy of the imbalance-based approach using the example of *ALK* fusion detection by analyzing the differences in the coverage levels of its tyrosine kinase and non-tyrosine kinase exons through RNAseq reads. For this, we analyzed 906 experimental whole transcriptome sequencing profiles from a pan-cancer cohort of samples. Based on the developed algorithm for determining *ALK* coverage asymmetry, we predicted the presence of *ALK* fusions in 13 samples. To validate the *ALK* status, we performed targeted sequencing using two NGS panels, and, where biomaterial was available, confirmed the results by Sanger sequencing. The predicted *ALK* fusions were confirmed in 11 out of 13 samples, giving a prediction accuracy of 96%. We also demonstrated that the *ALK* coverage asymmetry detection method worked with the panel sequencing data.

## 2. Materials and Methods

### 2.1. Biosamples and RNA Sequencing

Our own whole transcriptome data from 1039 cancer samples across 27 tumor types were used in this study. Most of the samples (*n* = 764) either were from an Oncobox clinical trial (Clinicaltrials.gov ID NCT03724097, NCT03521245) or were biosamples submitted for Oncobox molecular testing. For each patient biospecimen, written informed consent for participation in this study was obtained from the patient or their legal representative. The consent process and study design were guided and approved by the local ethics committees of the Vitamed Clinic (Moscow, Russia).

Patients’ clinical information, including ALK status in some cases, was previously collected.

RNA extraction, library preparation, sequencing, and RNAseq data processing were performed as described in [47,52], and the resulting BAM files were analyzed.

To minimize bias in NGS data, we used standardized protocols for RNA extraction and library preparation. RNA was extracted from formalin-fixed paraffin-embedded (FFPE) samples using the RNeasy FFPE Kit (Qiagen, Hilden, Germany) and from bone marrow nucleated cells using the RNeasy Kit (Qiagen, Hilden, Germany). Sequencing libraries were prepared using the strand-specific KAPA RNA HyperPrep Kit with RiboErase (Roche, Basel, Switzerland). NGS was performed on either the HiSeq 3000 (Illumina, San Diego, CA, USA) or the NextSeq 550 (Illumina, San Diego, CA USA) in single-end mode, with read lengths of 50 bp or 75 bp, respectively.

After excluding samples with less than 2.5 million unique reads, 906 profiles remained. A total of 119 samples (13.1%) represented NSCLC and 788 (86.9%) represented other tumor types (Table 1). The gender and age characteristics of all cohorts are shown in Table 1.

### 2.2. Exon Coverage Calculation

*ALK* is expressed at low levels in the majority of normal human tissues. In the event of *ALK* fusion with other genes, a segment on the 5′ end of *ALK* (pre-TK) was excluded from the resulting oncogene. Given that the fusion transcript exhibited a markedly elevated expression level relative to that of the wild-type *ALK* transcript, a greater number of reads would be generated from the fusion transcript and aligned with the TK-related exons of *ALK*. Accordingly, an *ALK* rearrangement could be predicted on the basis of a comparison of the expression of the 5′ and 3′ regions of the gene.

The input files were BAM (binary alignment/map) files resulting from whole transcriptome sequencing. Deduplication of aligned sequencing reads was performed using the MarkDuplicates module of the Picard Toolkit with default parameters. Coverage asymmetry of TK and non-TK related exons of *ALK* was calculated using the following algorithm. RNA sequencing reads were aligned to the human genome (hg38, version GCF_000001405.39) using STAR v 2.7.4a. For *ALK* coverage statistics, only reads with a mapping quality (MQ) > 100 and an alignment length of at least 10 bp were considered. This information was binned to the coordinates of *ALK* exons obtained from the Ensembl database (for ENST00000389048.8), and the coverage was normalized to the length of the corresponding exons. Additionally, for within-sample normalization, the coverage was divided by the total number of reads and multiplied by 1,000,000. The approach utilized for coverage normalization was analogous to the RPKM metric for gene expression calculation but was applied to individual exons rather than to entire genes.

Coverage was calculated considering the strand specificity of the reads separately for antisense (corresponding to *ALK* sense transcripts) and sense reads (corresponding to *ALK* antisense transcripts). The former were visualized on the plots as “positive” bars. *ALK* antisense transcript reads were plotted as “negative coverage” bars and only used for quality control.

To assess coverage asymmetry, the coverage levels of exons 2–6 (non-TK) and exons 20–24 (TK-related) were compared using a one-sided Mann–Whitney U test with the alternative hypothesis that the coverage of non-TK-related exons was lower than that of TK-related exons. The significance of the coverage difference was determined based on the *p*-value of the statistical test.

The Mann–Whitney U test was used due to the non-normality of the coverage distribution, which was assessed using the Shapiro–Wilk test with Benjamini–Hochberg correction for multiple hypothesis testing (with the null hypothesis that the distribution was normal).

The code used for the analysis and the accompanying data are available at https://github.com/smiranast/coverage_asymmetry (accessed on 5 November 2024).

The coverage depth of *ALK* exons 20–24 was also calculated as the number of bases of all reads matching these exons divided by their combined length.

### 2.3. Experimental Validation of ALK Fusion Transcripts by Targeted NGS

Two targeted NGS panels were used for *ALK* rearrangement verification: the TruSight RNA Fusion Panel (Illumina, San Diego, CA USA) and the OncoFu Elite (for RNA) Panel v1.0 (Nanodigmbio, Nanjing, China). NGS libraries were prepared according to the manufacturer’s protocol. For the TruSight panel, libraries were prepared by starting from previously extracted RNA samples. For the OncoFu panel, hybridization enrichment was performed on previously prepared cDNA libraries, which were used for whole transcriptome sequencing.

Sequencing was performed on the FASTASeq 300 platform (GeneMind Biosciences, Shenzhen, China) (2 × 75 bp paired end) with an expected yield of approximately 4.5 million reads per sample for the TruSight panel and 2.5 million reads per sample for the OncoFu panel.

### 2.4. Experimental Validation of ALK Fusion Transcripts by Sanger Sequencing

To generate amplicons for Sanger sequencing, single-stranded cDNA was synthesized using the MMLV RT kit (Evrogen, Moscow, Russia) according to the manufacturer’s instructions. Reverse transcriptase was pre-diluted ten-fold with enzyme dilution buffer. Reverse transcription was performed using gene-specific reverse primer for *ALK* exon 20 (5′-GCTTGCAGCTCCTGGTG-3′), with a final concentration of 500 nM.

For preparative PCR, the following reagents were used (all from Evrogen, Moscow, Russia): 10× Turbo Buffer, dNTP mix (10 mM each), HS-Taq DNA polymerase, 50× SYBR Green I, and PCR-grade water. PCR was performed in 50 uL volumes using a T100 Thermal Cycler (Bio-Rad, Hercules, CA USA). The thermal cycling protocol was (1) 2 min at 95 °C; (2) 40 cycles of 15 s at 95 °C, 20 s at 60 °C, and 10 s at 70 °C; and (3) 1 min at 70 °C. The primer pairs are listed in Table 2.

Amplicons were purified using the standard NGS Clean and Select Beads protocol (Meridian Bioscience, Cincinnati, OH, USA), with a twofold excess of magnetic bead suspension volume. Sanger sequencing was performed on the genetic analyzer 3500xL (Applied Biosystems, Foster City, CA, USA).

### 2.5. Bioinformatics Approach to Detecting ALK Fusion Transcripts

*ALK* fusion transcripts were identified in both RNAseq profiles and data from NGS panels using STAR-Fusion software [16] (version STAR-2.7.2b). The pre-assembled genome build GRCh38_gencode_v33_CTAT_lib_Apr062020.plug-n-play was used as the reference genome sequence. For STAR-Fusion, default parameters were used.

Fusion candidate files were generated, followed by extraction of the relevant RNA sequencing reads associated with *ALK*. The resulting data were then verified by manual inspection using UCSC BLAT and the UCSC Genome Browser (https://genome-euro.ucsc.edu, accessed 1 August 2024). Candidate *ALK* fusions were evaluated based on the following criteria: (i) whether the sequencing read spanned an exon junction between two distinct, previously characterized transcripts, (ii) whether the junction site corresponded precisely to exon boundaries of known genes, considering established splice sites, and (iii) whether both transcripts were aligned in the same orientation, indicating the presence of a putative fusion RNA. Reads meeting these criteria were considered as evidence of fusion.

### 2.6. Statistical Analysis

Survival analysis was performed using the Kaplan–Meier method and the ‘survival’ package of R (https://cran.r-project.org/package=survival, accessed on 16 September 2024) Kaplan–Meier curves were plotted using the ‘ggsurvplot’ function of ‘survminer’ package (https://cran.r-project.org/web/packages/survminer/index.html, accessed on 16 September 2024). Cox proportional hazard analysis for progression-free survival (PFS) was performed to assess differences in survival between these groups using the ‘coxph’ function of the ‘*survival*’ package of R (https://cran.r-project.org/package=survival, accessed on 16 September 2024). The hazard ratio (HR) and 95% confidence interval (CI) were calculated as the ratio of failure rates in patients with predicted *ALK* fusion relative to those without it. The statistical significance of differences between the groups was estimated using the log-rank test with the ‘logrank’ function from the Python 3 ‘scipy’ library [53] (https://docs.scipy.org/doc/scipy/reference/generated/scipy.stats.logrank.html, accessed on 16 September 2024). A *p*-value less than 0.05 was considered to be statistically significant.

## 3. Results

### 3.1. ALK Coverage Assymetry Screening

According to the COSMIC [20] (https://cancer.sanger.ac.uk/cosmic, accessed on 2 July 2024) and the FusionGDB 2.0 [21] (https://compbio.uth.edu/FusionGDB2/, accessed on 2 July 2024) databases, *ALK* rearrangements occur in intron 19 in approximately 92% of cases, in intron 18 in 1.5% of cases, and in exon 20 in approximately 4% of cases. Thus, in the case of *ALK* fusion, the expression levels of exons 20–29 are expected to be elevated, while the expression levels of exons 1–19 should be close to the control levels.

First, we investigated whether *ALK* coverage asymmetry could be detected in the RNAseq data. For this purpose, we analyzed our experimental dataset consisting of 906 WTS profiles from samples representing different malignant tumor types (Table 1). Initially, we determined the expression levels of all *ALK* exons and normalized coverage to the exon length and the total number of reads in the sample. As a criterion for asymmetry of *ALK* gene coverage, we used the *p*-value calculated by the Mann–Whitney U test, comparing the coverage levels of exons 20–24 (corresponding to the first half of the TK domain) with those of exons 2–6. Coverage was considered asymmetric if the *p*-value was less than 0.05. The number of selected exons (*n* = 5) in each region was justified by two considerations: (i) to reach the 5% significance level and (ii) to verify the functionality of a putative *ALK* fusion, as it was important to ensure that the TK domain was preserved. Thus, we selected the first five exons of the TK domain for the TK region coverage calculation and the same number of exons at the start of the gene, except for the first exon, which was expected to be artificially underrepresented due to the cDNA priming protocol used.

It is important to note that the RNAseq library preparation protocol we used was strand-specific, and only *ALK* transcript sense reads were used for the asymmetry calculation. This is important because in many samples, the 3′ end of *ALK* exons was significantly covered by *ALK* antisense reads (as shown in Supplementary Figure S1). These reads may belong to antisense transcripts involving fragments of the *ALK* gene that could be initiated by read-through downstream transcription of the *CLIP4* (CAP-gly domain containing linker protein family member 4) gene [54,55], which was expressed in the opposite orientation and located “tail to tail” only ~9 kb away from *ALK*.

**Figure 3:**
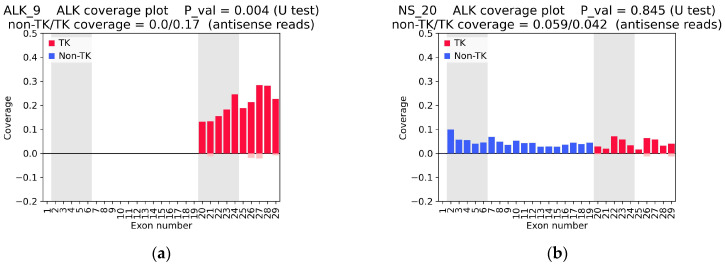
The *ALK* coverage plots are based on RNAseq data and normalized to the exon length and total number of reads in the sample. The coverage of the *ALK* sense reads is shown on a positive scale, while the *ALK* antisense reads are shown on a negative scale. (**a**) The ALK_9 sample showed pronounced coverage asymmetry and overexpression of exons 20–29. (**b**) The NS_20 sample showed uniformly high coverage of all exons. TK—tyrosine kinase domain-related exons; non-TK—exons not related to the tyrosine kinase domain; and non-TK/TK coverage—ratios of the mean coverage of five non-TK exons (exons 2–6) and five TK exons (exons 20–24).

A total of 13 samples from our experimental collection showed asymmetric coverage according to the above criteria, suggesting the presence of *ALK* fusion (Figure 3, Supplementary Figure S2). Of these, 12 represented lung cancer, and one represented ovarian cancer (Table 3).

We also found that in 79 samples (of which 62 were central nervous system (CNS) tumors, seven were ovarian cancers, five were NSCLC, and five were other cancers), all *ALK* exons were transcribed, indicating the increased expression of the wild-type gene. The expression of wt-*ALK* in a large number of CNS tumors was expected, as it is known that ALK is expressed in brain tissues [10]. Four examples of uniform *ALK* coverage (ALK_15, ALK_3, OC_7, and NS_20) are shown in Table 3 and Supplementary Figure S2.

We then compared these data with the available clinical information on *ALK* status. In nine cases, the prediction matched the clinical annotation of our experimental biospecimens. In two samples, ALK_3 (IHC-positive) and ALK_15 (showed conflicting IHC results from two laboratories), *ALK* exon coverage was uniform, indicating increased expression of the wild-type enzyme. Since IHC targeted the carboxy terminus of ALK, it could not distinguish between the expression levels of ALK fusions and wild-type enzymes. Another two samples (LuC_62 and LuC_68) were annotated as ALK-negative, but they were predicted to be *ALK* fusion carriers by our algorithm. Finally, three samples (ALK_6_2, ALK_14, and LuC_103) were annotated as ALK-positive, but no *ALK* expression was detected in the RNAseq data.

**Figure 4:**
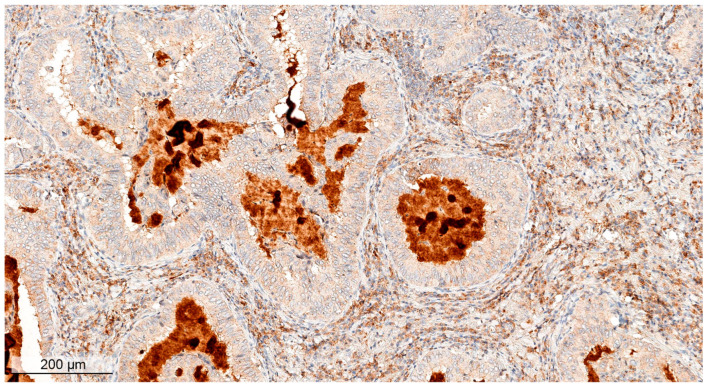
ALK immunostaining using clone D5F3 (Ventana) for sample LuC_103.

Unfortunately, paraffin-embedded tissue was only available for one case with discordance between the clinical annotation and prediction of *ALK* fusion based on an imbalance in RNAseq data, i.e., LuC_103. Upon review of the IHC (clone D5F3, Ventana, Oro Valley, AZ USA) results for this sample by a pathologist, it was determined that the ALK-positive status had previously been incorrectly assigned, as high signal foci were present in the normal mucosa while the tumor cells were unstained (Figure 4; additional high-resolution images are provided in Supplementary Figure S3). This apparent *ALK*-negative status was indirectly confirmed by the patient’s lack of response to the ALK-targeted therapeutic alectinib. Thus, we were able to algorithmically identify at least one false-positive clinical case.

Given the discrepancy between the clinical annotation of ALK status and the predicted *ALK* fusion status based on the coverage imbalance observed in a number of cases (ALK_3, ALK_6_2, ALK_14, ALK_15, LuC_62, LuC_68, and LuC_103), we proceeded to validate the *ALK* status through an experimental approach involving targeted sequencing, which offered high sensitivity and specificity. To this end, we used two panels specifically designed to detect fusion transcripts, the TruSight RNA Fusion Panel (Illumina, San Diego, CA, USA) and the OncoFu Elite (for RNA) Panel v1.0 (Nanodigmbio, Nanjing, China).

### 3.2. ALK Fusion Validation with Targeted NGS

For experimental validation, we selected a pool of 50 samples including all predicted *ALK* fusion cases (with *p*-value < 0.05 for coverage asymmetry; *n* = 13), all samples without coverage asymmetry but clinically annotated as ALK-positive (*n* = 6), and control samples without *ALK* coverage asymmetry and with negative or unknown clinical ALK status (*n* = 31), as shown in Supplementary Table S1.

Both targeted NGS panels used for experimental validation were based on hybridization enrichment. For the OncoFu panel, previously prepared cDNA libraries were used as the starting material. For the TruSight panel, libraries were prepared de novo from RNA samples. The STAR-Fusion package [16] was used to search for fusion reads in the resulting panel sequencing data. STAR-Fusion was also used to analyze the WTS data for junction reads confirming *ALK* fusions.

The main results of the panel sequencing experiments are shown in Table 4; more detailed information is provided in Supplementary Table S1. Junction reads for different variants of the *EML4::ALK* were detected in the WTS data for only four samples. Using panel sequencing, *ALK* fusions were detected in 11 samples. Consistent with previously published studies, the most common variants were found to be *EML4::ALK* fusions with a breakpoint in *EML4* intron 6 (variant 3) and intron 13 (variant 1) [23,56,57]. One sample (ALK_16) showed a rearrangement in *EML4* in intron 20 (variant 2).

Notably, the results from the above two fusion panels showed some variation: the OncoFu panel generally had more supporting reads; in two samples, the TruSight panel did not detect the fusion while the OncoFu panel did, and in one sample, the reverse was observed (Table 4). The elevated number of supporting reads in OncoFu was not attributable to the presence of duplicate reads, as the data were analyzed using the STAR-Fusion package, which exclusively analyzed unique reads. Since the full protocol details for OncoFu and TruSight panels were unknown, we assumed that differences in the number of chimeric reads could result from: (i) different panel sizes, which affected the coverage of each individual target (the TruSight panel included 507 genes, while OncoFu included only 105 genes); (ii) differences in the design of probes used for enrichment; (iii) differences in library preparation protocols: for enrichment with OncoFu probes, ready-made cDNA libraries were used, which were prepared following the KAPA RNA HyperPrep Kit with RiboErase (Roche, Switzerland), whereas the TruSight panel required de novo library preparation using Illumina reagents, which were prepared following the manufacturer’s instructions; and (iv) other factors such as the composition of hybridization and wash buffers.

In clinical practice, parallel testing of the sample with two panels is unlikely due to the cost of analysis and limited sample material, making conflicting results from targeted sequencing improbable. However, cases of conflicting results from other fusion tests, such as IHC and FISH, are relatively common. In these cases, re-evaluation or an alternative test (e.g., PCR for common fusion variants) may be ordered, or, if the material is limited, the physician’s decision regarding targeted therapy is based on professional society guidelines and personal experience. Since no single method provides 100% sensitivity or specificity on a large scale, the most reliable approach is to use orthogonal methods.

In all 11 targeted NGS-positive samples, the presence of the *ALK* fusion was predicted by our algorithm based on the assessment of *ALK* coverage asymmetry. In all samples where the algorithm predicted the absence of the fusion (*n* = 37), the fusion was not detected by panel sequencing. In two samples (LuC_68 and OC_25), the *ALK* coverage imbalance was statistically significant; however, *ALK* fusion was not confirmed in any of the targeted fusion detection panels. A review of the reads mapping to the *ALK* exons revealed a markedly low coverage depth for four samples: ALK_1_2, ALK_4, LuC_68, and OC_25 (Supplementary Table S1). Of these, the panels were able to detect fusion in two samples (ALK_1_2 and ALK_4) with an extremely low number of supporting reads. This finding suggested that low coverage depth could contribute to the generation of false positive predictions according to the coverage imbalance analysis (see Section 3.3).

Subsequently, we validated the fusion findings from the NGS panels in five samples (ALK_5, ALK_8, ALK_9, ALK_10, and ALK_16), in which sufficient RNA had been retained for further experimental examination. To this end, we employed Sanger sequencing. This analysis confirmed the corresponding *EML4::ALK* fusion variants in all tested samples (Supplementary Figure S4).

Compared to the targeted NGS results, our *ALK* fusion prediction algorithm using RNAseq coverage asymmetry demonstrated 96% accuracy, with 100% sensitivity and 94.9% specificity. It was proposed that the specificity of the method could be further increased by the introduction of an additional parameter, namely, the depth of TK domain coverage (Section 3.3). Of course, these data were preliminary as we only had a small number of ALK-positive samples available to test our approach, and the approach needed to be validated in larger cohorts of true positive samples.

### 3.3. Minimum Required RNAseq Coverage Depth for ALK Asymmetry Analysis

Given the discrepancies observed between different ALK fusion detection methods for low-coverage samples (LuC_68, OC_25, ALK_1_2, and ALK_4), we sought to determine the minimum coverage of RNAseq reads at which the prediction of the presence of an ALK fusion by asymmetry could be considered reliable. Since we did not have enough true positive samples to experimentally determine a coverage depth threshold at the time, we used the subsampling method to model how coverage affects assay detection. From the original FASTQ files for three true-positive samples with confirmed *ALK* fusions (ALK_2, ALK_5, and ALK_16), as well as for two samples expressing wt-*ALK* (ALK_15 and NS_20), we randomly selected 1/2/5/10/20 million reads in triplicate for each sample and each read count. For each subsample, we calculated the coverage depth of *ALK* exons 20–24 and the *p*-value (U-test) for *ALK* coverage asymmetry. Since the common factor for the detection of fusions and wild-type enzymes is the expression of the TK domain, the coverage depth of *ALK* exons 20–24 was chosen as the coverage metric. Supplementary Figure S5 shows the change in the *p*-value as a function of the *ALK* coverage. It can be seen that in true positive samples (ALK_2 and ALK_5), when simulating a TK coverage depth below 0.7, the *p*-value exceeded the statistical significance threshold in some cases, while in the true negative sample NS_20, at a coverage of about 0.2, an *ALK* asymmetry was erroneously detected. Consequently, it was essential to consider coverage depth when estimating asymmetry. The data obtained permitted a tentative estimation of the required minimum coverage depth for *ALK* exons 20–24, which was estimated to be 0.7.

### 3.4. ALK Coverage Asymmetry in Targeted NGS Data

Both the TruSight panel and the OncoFu panels were based on hybridization probe enrichment but differ in the number of target genes (507 versus 102, respectively). In both panels, probes covered all exons of *ALK*, which, in principle, made it possible to assess *ALK* coverage asymmetry based on panel sequencing data as well. We tested this assumption. In both panels, *ALK* coverage asymmetry was indeed clearly evident in the cases with confirmed fusions. However, the TruSight data were of greater interest because, in the case of OncoFu, the enrichment was performed on pre-prepared RNAseq libraries, and the coverage plots fully mirrored those obtained for WTS. Conversely, for TruSight sequencing, libraries were freshly prepared from RNA. Thus, they could serve as an independent confirmation of the *ALK* coverage asymmetry observed in WTS.

**Figure 5:**
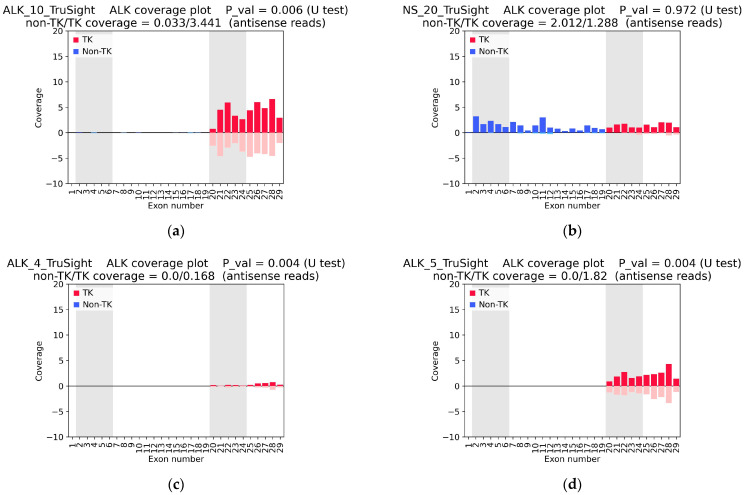

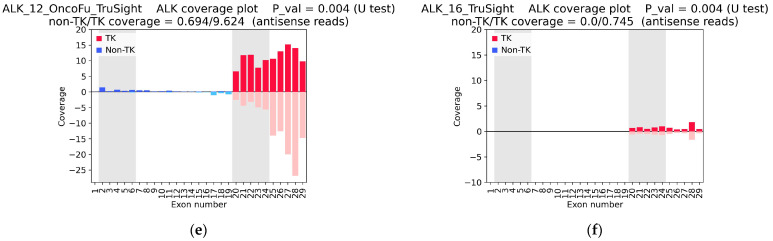
*ALK* coverage plots based on targeted RNAseq data, obtained with the TruSight panel, normalized to exon length and the total number of reads in the sample. Coverage of *ALK* sense reads is shown on a positive scale, while *ALK* antisense reads are shown on a negative scale. (**a**) The ALK_10 sample demonstrates clear *ALK* coverage asymmetry and a high number of fusion-supporting reads. (**b**) The NS_20 sample shows the detectable expression of all *ALK* exons. (**c**–**f**) ALK_4, ALK_5, ALK_12, and ALK_16 samples, respectively, with *ALK* coverage asymmetry but very few (or no) fusion-supporting reads. TK—tyrosine kinase domain-related exons; non-TK—exons not related to the kinase domain; and non-TK and TK coverage—mean coverage of five non-TK exons (exons 2–6) and of five TK exons (exons 20–24), respectively.

Figure 5 shows individual *ALK* coverage plots based on TruSight data. Results for all samples are shown in Supplementary Figure S6. For all samples with confirmed *ALK* fusion, a clear coverage asymmetry was observed in the TruSight data (with *p*-value < 0.01). Furthermore, the TruSight data reproduced the uniform coverage pattern of all *ALK* exons in samples ALK_3, ALK_15, and NS_20 observed in the WTS data, indicating the expression of wild-type *ALK* (Figure 3b and Figure 5b). Most interestingly, the samples with a low number of fusion-supporting reads in the TruSight NGS data (ALK_1_2, ALK_2, ALK_4, ALK_5, ALK_12, and ALK_16) showed a clear heterogeneity in *ALK* coverage with significantly increased expression levels of exons 20–29 (Figure 5c–f).

An important implication of this is that NGS panel data could be used to search for potential *ALK* fusions, regardless of the presence of supporting junction reads. *ALK* coverage asymmetry could also be used as an additional confirmation criterion.

### 3.5. ALK Coverage Asymmetry in RNAseq Data and Response to Targeted Therapy

In our cohort there were 13 patients with ALK-positive status by clinical annotation and one with controversial IHC results (ALK_15). Of these 14 samples, ten patients listed in Table 5 received ALK-targeted therapy. The rest (ALK_5, ALK_6_2, ALK_14, and ALK_15) either did not receive targeted therapy for various reasons or were lost during follow-up, and data on the efficacy of therapy were unknown.

**Figure 6:**
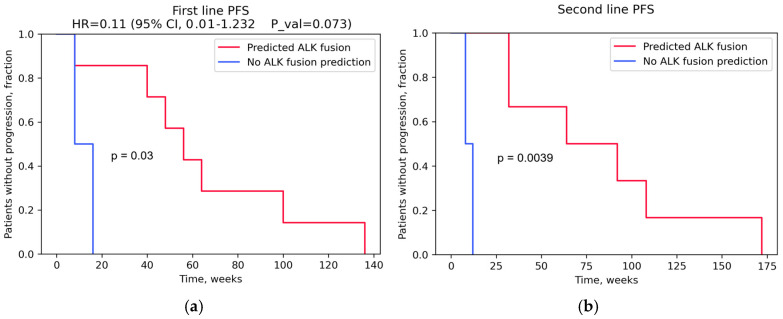
Kaplan–Meier plots for PFS data in lung cancer patients receiving ALK-specific targeted therapies: (**a**) for first-line ALK-specific therapy and (**b**) for second-line ALK-specific therapy. PFS—progression-free survival; HR—hazard ratio; CI—confidence interval; and P_val—*p*-value.

Crizotinib and ensartinib were used in the first-line setting, and alectinib, brigatinib, and crizotinib were used in the second-line setting. Information on therapeutic agents and progression-free survival (PFS) is presented in Table 5. Kaplan–Meier plots for PFS are shown in Figure 6 for patients with predicted *ALK* fusion based on *ALK* coverage asymmetry in RNAseq data and for patients without *ALK* coverage asymmetry. The former group showed significantly longer PFS in response to TKI therapy (*p* < 0.05). A statistically significant determination of the hazard ratio was not possible due to the small size of the comparison groups. However, the trend observed suggested that our method had the potential to predict the individual patient response to ALK-specific targeted therapies. A study with a larger group of patients was needed to establish the predictive power of our approach in more detail.

## 4. Discussion

Genetic methods for the analysis of tumor molecular markers are becoming increasingly widespread for two main reasons: (i) they allow the analysis of a large number of biomarkers simultaneously and (ii) their results are less susceptible to subjective interpretation compared to classical methods, such as IHC and FISH. For the identification of clinically significant gene fusions, targeted NGS panels are increasingly being used, demonstrating high sensitivity and specificity [58,59]. Since about 2020, the use of large multigene NGS panels for tumor marker analysis has been included in the guidelines of relevant professional communities [46,60]. Remarkably, during targeted TKI therapy, patients who tested positive for ALK fusions by NGS showed improved disease control rates and prolonged progression-free survival compared to NGS-negative patients, while IHC and FISH were less predictive [30].

Whole transcriptome sequencing (WTS), while primarily a scientific tool, also has great potential for implementation in clinical practice, as it allows the evaluation of a significantly larger number of cancer features compared to targeted NGS [61,62,63]. Unfortunately, WTS is less suitable for the detection of chimeric transcripts due to its limited coverage of potential fusion junctions.

Existing bioinformatic methods to detect fusions in WTS data rely on the identification of chimeric reads, i.e., reads that map to two different genomic regions. During library preparation, particularly at the adapter ligation stage, unintended ligation of random cDNA fragments can occur, resulting in artifact chimeric reads. To distinguish true fusion reads from such artifacts, various filtering criteria are applied, such as assessing gene orientation, preserving the reading frame, and checking for the occurrence of similar fusions in databases [35]. Several programs have been developed for this purpose, including deFuse [64], FusionCatcher [65], PRADA [66], FusionHunter [67], SOAPfuse [68], JAFFA [69], STAR-Fusion [70], and Arriba [71]. STAR-Fusion and Arriba algorithms have been found to be the most accurate in identifying fused transcripts in cancer [16,72,73]. However, the number of junction reads is typically low, even in targeted sequencing, as shown in this study. In addition, even when true chimeric reads are present, the output of fusion detection programs often includes multiple false-positive “garbage” results after all filtering, making the analysis much more difficult.

At the same time, our proposed approach for predicting *ALK* gene fusions using RNAseq exon coverage analysis does not require direct identification of the fusion junction. Instead, the presence of a gene fusion can be predicted by analyzing the uniformity of expression of the target gene exons. As shown in our study, this method works well to detect *ALK* fusions using WTS data with an accuracy of 96% (sensitivity 100%, specificity 94.9%). We also demonstrate that coverage asymmetry can be used to detect *ALK* fusions in panel sequencing data, allowing the identification or confirmation of rearrangements for which no supporting reads are found. In addition, in a small sample of patients receiving ALK-targeted therapy, the proposed algorithm is shown to be potentially successful in predicting treatment response.

According to our preliminary data, published in [47], as well as unpublished data, the proposed RNAseq approach can also identify fusions with several other tyrosine kinases involved as 3′ partners of chimeric genes. Based on the analysis of coverage unevenness, gene fusions are suspected in four samples for *RET*, two samples for *ROS1*, and one sample for *PDGFRB*, and the presence of the corresponding fusions in these samples is then confirmed by panel sequencing. However, we have not yet collected enough positive samples to evaluate the predictive accuracy in this case. Nevertheless, our RNAseq data are consistent with previous findings in *ROS1*, *RET*, and *PDGFR* fusion-positive samples where 5′/3′ asymmetry is detected using PCR-based [50] and NanoString-based methods [51].

In comparison to alternative methodologies, the proposed algorithm offers the following benefits: (1) it enables the prediction of a fused transcript exclusively through the calculation of *ALK* exon coverage, even in the absence of chimeric read detection, in contrast to other approaches for the analysis of RNAseq data; (2) it identifies only transcriptionally active chimeric genes, excluding passenger mutations, in contrast to FISH and DNA-based NGS; (3) it permits the evaluation of the integrity of the kinase domain within the chimeric gene, which is not possible with FISH or IHC; and (4) it allows the discrimination between the expression of the fused gene and the wild-type enzyme, unlike IHC. The main drawbacks of whole transcriptome RNAseq remain its cost and the greater complexity of data interpretation, and for these reasons, it is still not widely adopted in clinical practice. Thus, our approach cannot yet replace existing methods for fusion detection, but it allows for more clinically relevant information to be extracted from RNAseq data when it is performed for any reason.

It is important to emphasize that, although our study yielded promising results, further investigations using larger and more diverse datasets are essential to accurately determine the robustness and broader applicability of this approach. One parameter that needs to be elucidated is the threshold coverage level at which *ALK* fusion detection based on coverage asymmetry can be determined with a reasonable degree of confidence. In our simulation, reducing the coverage depth below 0.7 for the TK domain increases the likelihood of false negative and false positive results. Therefore, results obtained for samples with extremely low *ALK* coverage should be interpreted with caution.

Another limitation of our approach is that it does not allow for the identification of the 5′ partner in the fusion or the exact breakpoint of the 3′ partner (although it can be inferred from the exon expression pattern). Routine methods such as IHC share this limitation. Since anti-ALK targeted therapy specifically targets the ALK TK domain, clinical guidelines for its prescription do not require knowledge of the 5′ fusion partner gene [60]. While this does not limit the clinical application of our approach, it may limit the investigation of new fusion variants and their biological characteristics.

In summary, we hope that the current approach can be seen as an important new direction in RNA-based molecular cancer diagnostics for *ALK* and other oncogenic genome rearrangements.

## 5. Conclusions

Genetic methods for tumor molecular marker analysis are increasingly favored because of their ability to assess multiple biomarkers simultaneously and reduce subjective interpretation compared to traditional techniques such as IHC and FISH. While whole transcriptome sequencing (WTS) holds promise for a more comprehensive assessment of cancer characteristics, it faces challenges in detecting chimeric transcripts due to low true fusion read counts and potential false positives. Our proposed RNAseq approach, which predicts *ALK* gene fusions by exon coverage analysis without requiring identification of the fusion junction, achieves 96% accuracy (100% sensitivity, 94.9% specificity) in a limited cohort of ALK-positive tumors. Based on our preliminary data, the approach can also identify other tyrosine kinase fusions for *ROS1*, *RET*, and *PDGFRB*.

Despite limitations such as the inability to determine 5′ fusion partners, our method represents a significant advance in RNAseq molecular diagnostics for *ALK* and other oncogenic rearrangements. However, further investigation with larger and more diverse datasets is essential to accurately determine the robustness and broad applicability of this approach.

## Figures and Tables

**Table 1:** Clinical characteristics of the included cancer cases.

Cancer Type ^1^	Total Cases Sequenced, *n* (%)	Gender, Male/Female	Age of Onset		
			Mean	Median	Range
NSCLC	119 (13.1%)	61.3%/36.1% ^2^	60.01	60	29–81
CRC	113 (12.5%)	46.0%/54.0%	56.97	57	32–85
TC	107 (11.8%)	20.6%/72.9% ^2^	47.69	50	11–77
BC	93 (10.3%)	0%/100%	51.54	52	27–81
CNS	73 (8.1%)	58.9%/35.6% ^2^	43.90	48	3–70
MM	56 (6.2%)	55.4%/44.6%	58.66	60	29–78
AL	50 (5.5%)	54%/46%	5.79	5	1–15
SC	45 (5.0%)	55.6%/44.4%	55.80	55	31–79
OC	44 (4.9%)	0%/100%	48.95	47	27–80
PC	35 (3.9%)	51.4%/45.7% ^2^	59.67	62	35–77
KC	32 (3.5%)	62.5%/37.5%	55.16	55	40–69
Other	139 (15.3%)	43.2%/55.4% ^2^	52.21	54	12–84

^1^ Cancer type abbreviations: NSCLC—non-small cell lung cancer; CRC—colorectal cancer; TC—thyroid cancer; BC—breast cancer; CNS—central nervous system cancer; MM—multiple myeloma; AL—acute lymphoblastic/myeloid leukemia; OC—ovarian cancer; SC—stomach cancer; PC—pancreatic cancer; KC—kidney cancer; and SCLC—small cell lung cancer. ^2^ For the remaining several samples, the annotation was lost.

**Table 2:** List of primers used to amplify the fusion transcript fragment around the junction point.

Target Fusion	Forward Primer, 5′–3′	Reverse Primer, 5′–3′
*EML4(13)::ALK(20)*	TGGAGATGTTCTTACTGGAGACTC	GCTTGCAGCTCCTGGTG
*EML4(20)::ALK(20)*	CATCACACACCTTGACTGGTC	GCTTGCAGCTCCTGGTG

**Table 3:** Characteristics of putative *ALK* fusions identified by exon coverage asymmetry.

Sample ID	Sex	Age on Onset	Cancer Type	ALK Status (Clinical Data) ^1^	RNAseq Data	
					ALK Coverage Asymmetry, *p*-Value ^2^	Mean Coverage of *ALK* ex2-6/ex20-24 ^3^
ALK_1_2	M	56	Lung adenocarcinoma	ALK+ (IHC)	0.036	0.0/0.018
ALK_2	F	48	Lung adenocarcinoma	*EML4(6)::ALK(20)* (RT-qPCR)	0.004	0.0/0.080
ALK_3	F	60	Lung adenocarcinoma	ALK+ (IHC)	0.971	0.023/0.007
ALK_4	M	52	Lung adenocarcinoma	ALK+ (FISH)	0.013	0.0/0.012
ALK_5	F	48	Lung adenocarcinoma	ALK+ (FISH)	0.005	0.002/0.103
ALK_6_2	F	66	Lung adenocarcinoma	ALK+ (FISH)	1	0.0/0.0
ALK_8	M	46	Lung adenocarcinoma	ALK+ (IHC), *EML4(13)::ALK(20)* (RT-qPCR)	0.006	0.003/0.138
ALK_9	F	51	Lung adenocarcinoma	ALK+ (FISH)	0.004	0.0/0.170
ALK_10	M	45	Lung adenocarcinoma	ALK+ (RT-qPCR, FISH)	0.004	0.0/0.206
ALK_12	F	42	Lung adenocarcinoma	ALK+ (IHC, FISH)	0.037	0.002/0.023
ALK_14	M	64	Lung adenocarcinoma	ALK+ (IHC, FISH)	0.949	0.004/0
ALK_15	M	53	Lung adenocarcinoma	ALK+/− (controversial IHC results in two labs)	0.925	0.203/0.117
ALK_16	F	29	Lung adenocarcinoma	ALK+ (IHC, FISH)	0.005	0.002/0.158
LuC_62	F	62	Lung adenocarcinoma	ALK− (method unspecified)	0.004	0.0/0.038
LuC_68	M	59	Lung adenocarcinoma	ALK− (IHC; barely visible signal)	0.004	0.0/0.007
LuC_103	M	75	Lung adenocarcinoma	ALK+ (IHC) ^4^	1	0.0/0.0
LuC_104	M	46	Squamous cell carcinoma	unknown	0.004	0.0/0.125
NS_20	M	58	Glioblastoma	unknown	0.845	0.059/0.042
OC_25	F	58	Ovarian cancer	unknown	0.006	0.001/0.013

^1^ ALK status determined by FISH or IHC or RT-qPCR. ^2^ The *p*-value was calculated using the Mann–Whitney U test for *ALK* exons 20–24 versus exons 2–6. ^3^ The mean coverage levels for *ALK* exons 20–24 and exons 2–6 were calculated based on *ALK* sense reads only. ^4^ The IHC status for LuC_103 was reviewed after the NGS results were obtained.

**Table 4:** Results of *ALK* fusion validation using the TruSight and OncoFu targeted NGS panels.

Sample ID	Clinical ALK Status	RNAseq Coverage-Predicted *ALK* Fusion ^1^	*ALK* Fusion Found in RNAseq (Number of Junction Reads)	TruSight Results (Number of Junction+Spanning Reads)	OncoFu Results (Number of Junction+Spanning Reads)
ALK_1_2	positive	yes	no	*EML4(6)::ALK(20)* (1+0)	no fusion
ALK_2	positive	yes	no	*EML4(6)::ALK(20)* (1+0)	*EML4(6)::ALK(20)*? (0+3)
ALK_3	positive	no	no	no fusion	no fusion
ALK_4	positive	yes	no	no fusion	*EML4(6)::ALK(20)*? (0+3)
ALK_5	positive	yes	no	*EML4(13)::ALK(20)*? (0+1)	*EML4(13)::ALK(20)* (51+37)
ALK_6_2	positive	no	no	no fusion	no fusion
ALK_8	positive	yes	*EML4(13)::ALK(20)* (1)	*EML4(13)::ALK(20)* (1+4)	*EML4(13)::ALK(20)* (109+91)
ALK_9	positive	yes	*EML4(13)::ALK(20)* (2)	*EML4(13)::ALK(20)*? (0+8)	*EML4(13)::ALK(20)* (241+255)
ALK_10	positive	yes	*EML4(13)::ALK(20)* (5)	*EML4(13)::ALK(20)*? (0+24)	*EML4(13)::ALK(20)* (185+325)
ALK_12	positive	yes	no	*EML4(6)::ALK(20)* (2+0)	*EML4(6)::ALK(20)* (14+17)
ALK_14	positive	no	no	no fusion	no fusion
ALK_15	controversial	no	no	no fusion	no fusion
ALK_16	positive	yes	*EML4(20)::ALK(20)* (4)	no fusion	*EML4(20)::ALK(20)* (11+0)
LuC_62	negative	yes	no	*EML4(6)::ALK(20)* (2+1)	*EML4(6)::ALK(20)* (12+7)
LuC_68	negative	yes	no	no fusion	no fusion
LuC_103	positive	no	no	no fusion	no fusion
LuC_104	unknown	yes	no	*EML4(13)::ALK(20)* (3+1)	*EML4(13)::ALK(20)* (52+26)
NS_20	unknown	no	no	no fusion	no fusion
OC_25	unknown	yes	no	no fusion	no fusion

^1^ Prediction was made by *ALK* coverage asymmetry (considered positive, if *p* < 0.05).

**Table 5:** Response to TKI therapy and time to progression in lung cancer cases with ALK-positive clinical status.

Sample ID	IHC/FISH/PCR ALK Status	Predicted *ALK* Fusion ^1^	Validated *ALK* Fusion ^2^	First Line TKI	First Line PFS, Weeks	Second Line TKI	Second Line PFS, Weeks
ALK_1_2	positive	yes	yes	crizotinib	100	alectinib	unknown
ALK_2	positive	yes	yes	ensartinib	136	alectinib	64
ALK_3	positive	no	no	crizotinib	8	brigatinib	8
ALK_4	positive	yes	yes	crizotinib	8	alectinib	32
ALK_8	positive	yes	yes	crizotinib	64	alectinib	32
ALK_9	positive	yes	yes	unknown	unknown	alectinib	92
ALK_10	positive	yes	yes	crizotinib	56	alectinib	unknown
ALK_12	positive	yes	yes	crizotinib	48	brigatinib	108
ALK_16	positive	yes	yes	crizotinib	40	crizotinib	172
LuC_103	positive	no	no	crizotinib	16	alectinib	12

^1^ Predicted by *ALK* coverage asymmetry (positive when *p*-value < 0.05). ^2^ Validated with targeted NGS panels designed to capture driver fusion transcripts of known oncogenes. IHC—immunohistochemistry; FISH—fluorescent in situ hybridization; TKI—tyrosine kinase inhibitor; and PFS—progression-free survival.

## Data Availability

Data are contained within the article and supplementary materials.

## Supplementary Materials

The following supporting information can be downloaded at https://www.mdpi.com/article/10.3390/cancers16223851/s1. Figure S1: RNAseq reads mapping on *ALK* exons 20–29; Figure S2: *ALK* coverage plots based on RNAseq data; Figure S3: ALK immunostaining with clone D5F3 (Ventana) for sample LuC_103; Figure S4: Results of *ALK* fusion validation by Sanger sequencing; Figure S5: Dependence of statistical significance for *ALK* coverage asymmetry on the coverage depth of TK-related exons 20–24; Figure S6: *ALK* coverage plots based on TruSight panel data; Table S1: Clinical annotation, RNAseq *ALK* coverage asymmetry data, and the results of *ALK* fusion validation using targeted NGS panels TruSight and OncoFu for tumor samples from validation cohort.
