# Supplementary material for: A New Approach of Detecting ALK Fusion Oncogenes by RNA Sequencing Exon Coverage Analysis
Source: Cancers (Basel). 2024 Nov 16;16(22):3851. doi: 10.3390/cancers16223851 (PMC11592821; doi:10.3390/cancers16223851)
Supplement: Supplementary file 1 [file cancers-16-03851-s001.zip › Cancers_ALK_Supplementary Figures_S1-6_20241101.pdf]

Article "A new approach of detecting *ALK* fusion oncogenes by RNA sequencing exon coverage analysis"

Supplementary Figures

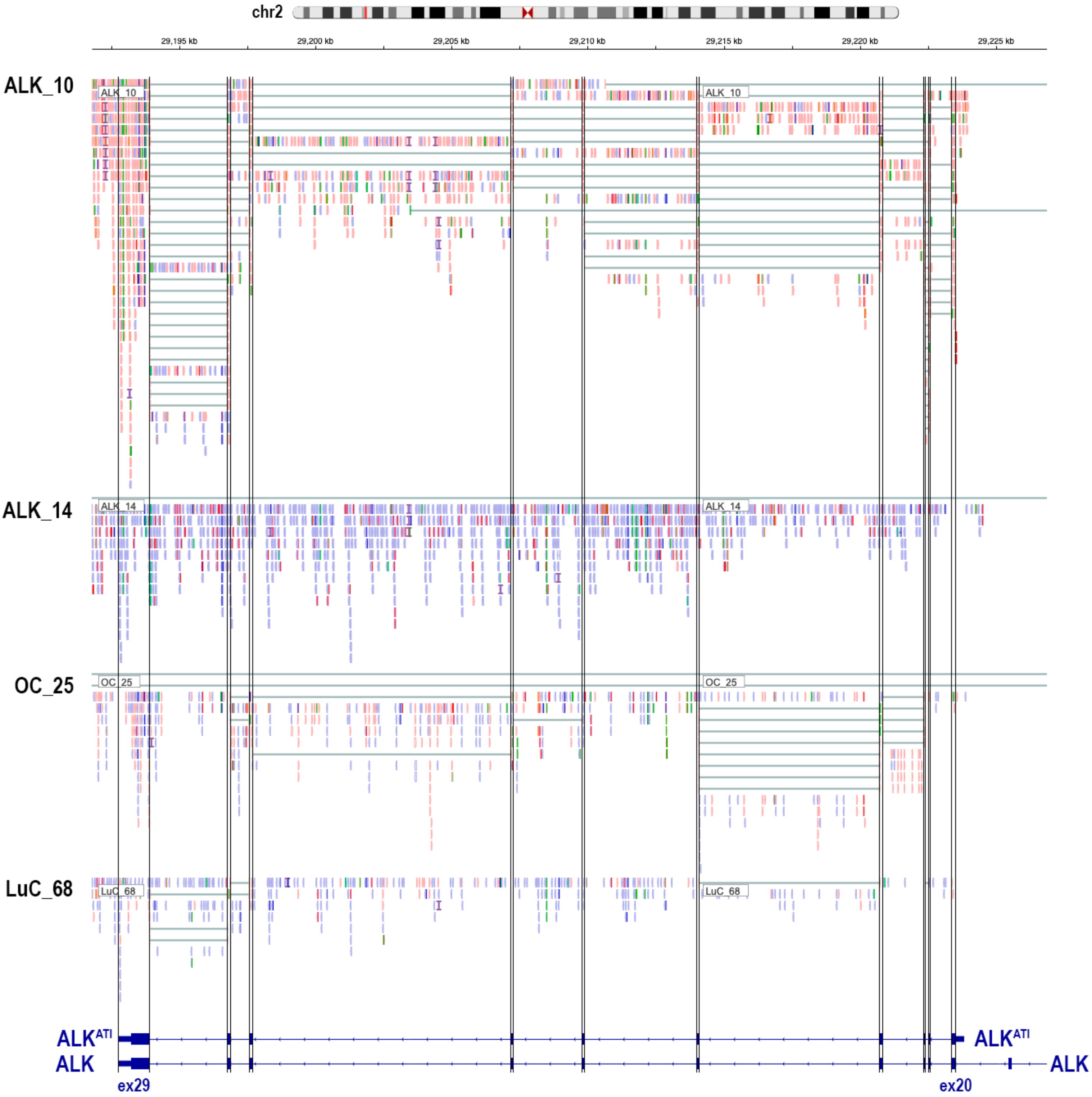

Figure S1. Examples of RNA-seq reads mapping to 3′-end of the *ALK* gene. Antisense reads (corresponding to *ALK*-sense transcripts) are shown in red, and sense reads (corresponding to *ALK*-antisense transcripts, most likely the downstream-of gene for the CLIP4 gene) are shown in blue. A notable proportion of antisense reads mapping to intronic regions are associated with the utilization of an rRNA depletion-based protocol for cDNA library preparation, which is suitable for highly fragmented RNA from FFPE samples. This results in the presence of a substantial fraction of immature mRNA in the NGS libraries.

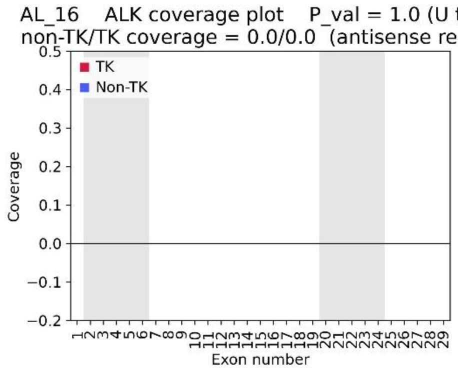
AL_16 ALK coverage plot P_val = 1.0 (U test)
non-TK/TK coverage = 0.0/0.0 (antisense reads)
TK
Non-TK
Coverage
Exon number

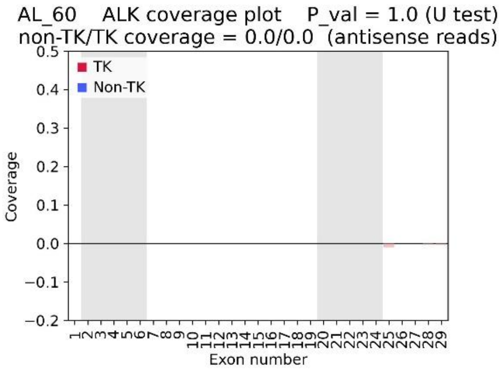
AL_60 ALK coverage plot P_val = 1.0 (U test)
non-TK/TK coverage = 0.0/0.0 (antisense reads)
TK
Non-TK
Coverage
Exon number

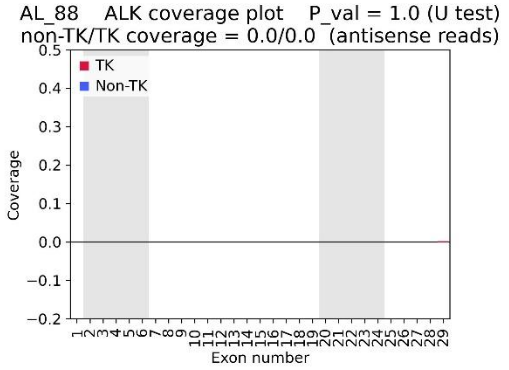
AL_88 ALK coverage plot P_val = 1.0 (U test)
non-TK/TK coverage = 0.0/0.0 (antisense reads)
TK
Non-TK
Coverage
Exon number

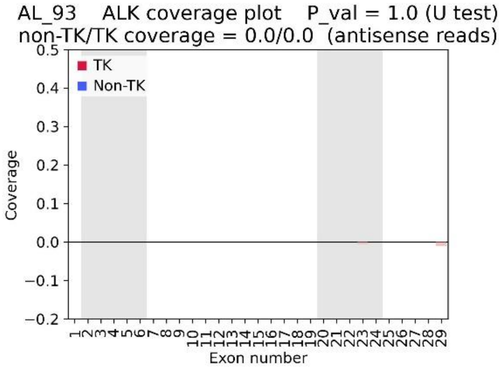
AL_93 ALK coverage plot P_val = 1.0 (U test)
non-TK/TK coverage = 0.0/0.0 (antisense reads)
TK
Non-TK
Coverage
Exon number

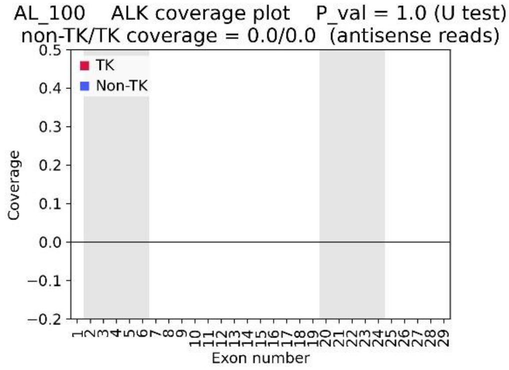
AL_100 ALK coverage plot P_val = 1.0 (U test)
non-TK/TK coverage = 0.0/0.0 (antisense reads)
TK
Non-TK
Coverage
Exon number

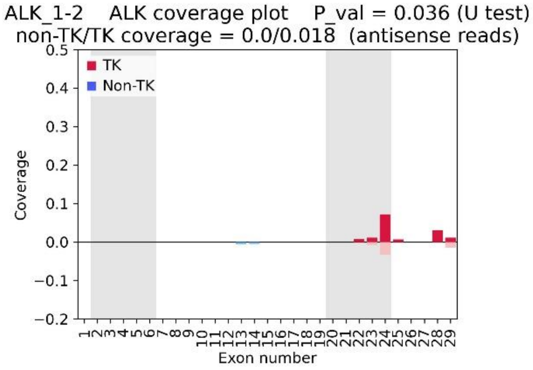
ALK_1-2 ALK coverage plot P_val = 0.036 (U test)
non-TK/TK coverage = 0.0/0.018 (antisense reads)
TK
Non-TK
Coverage
Exon number

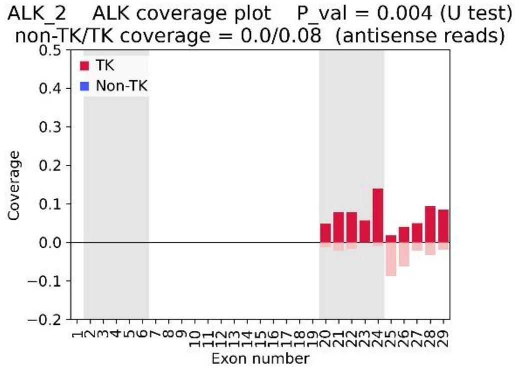
ALK_2 ALK coverage plot P_val = 0.004 (U test)
non-TK/TK coverage = 0.0/0.08 (antisense reads)
TK
Non-TK
Coverage
Exon number

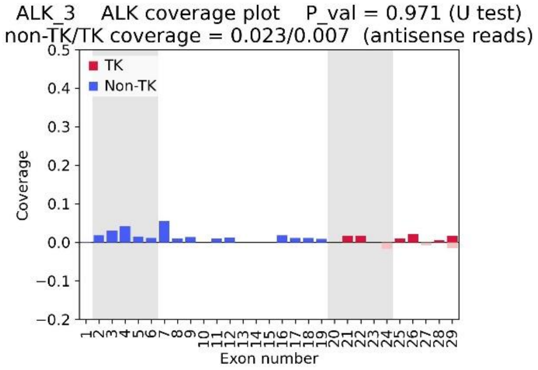
ALK_3 ALK coverage plot P_val = 0.971 (U test)
non-TK/TK coverage = 0.023/0.007 (antisense reads)
TK
Non-TK
Coverage
Exon number

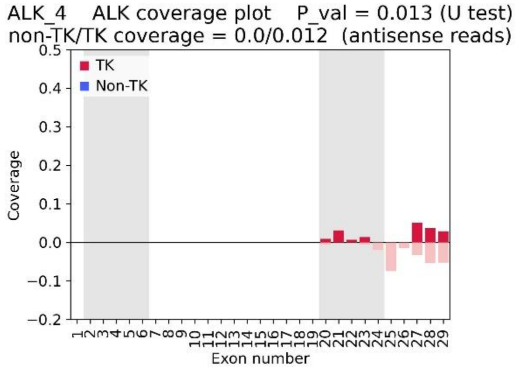
ALK_4 ALK coverage plot P_val = 0.013 (U test)
non-TK/TK coverage = 0.0/0.012 (antisense reads)
TK
Non-TK
Coverage
Exon number

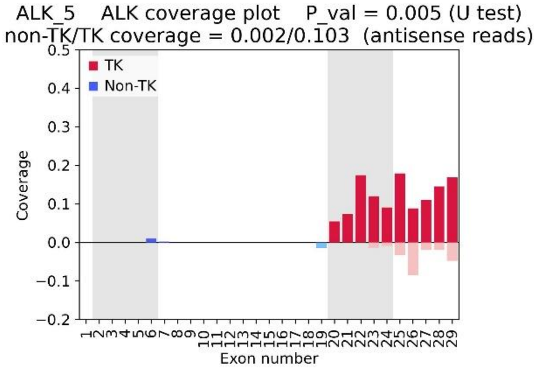
ALK_5 ALK coverage plot P_val = 0.005 (U test)
non-TK/TK coverage = 0.002/0.103 (antisense reads)
TK
Non-TK
Coverage
Exon number

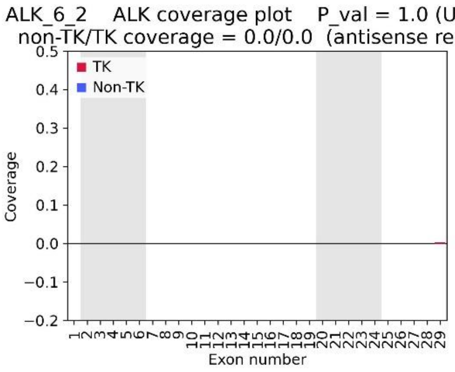
ALK_6_2 ALK coverage plot P_val = 1.0 (U test)
non-TK/TK coverage = 0.0/0.0 (antisense reads)
TK
Non-TK
Coverage
Exon number

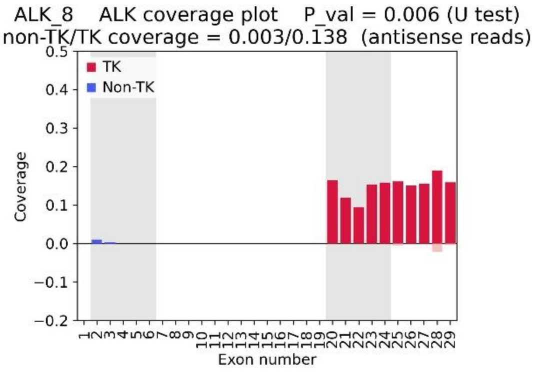
ALK_8 ALK coverage plot P_val = 0.006 (U test)
non-TK/TK coverage = 0.003/0.138 (antisense reads)
TK
Non-TK
Coverage
Exon number

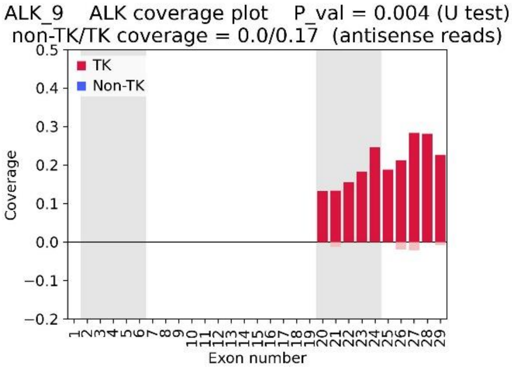
ALK_9 ALK coverage plot P_val = 0.004 (U test)
non-TK/TK coverage = 0.0/0.17 (antisense reads)
TK
Non-TK
Coverage
Exon number

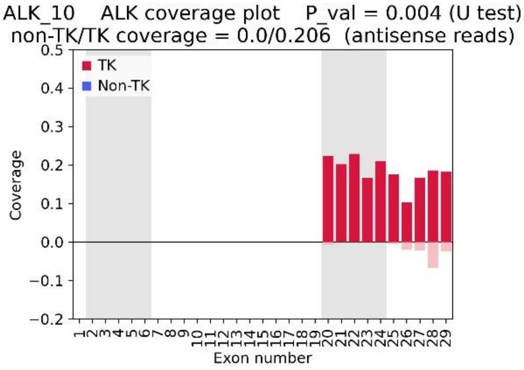
ALK_10 ALK coverage plot P_val = 0.004 (U test)
non-TK/TK coverage = 0.0/0.206 (antisense reads)
TK
Non-TK
Coverage
Exon number

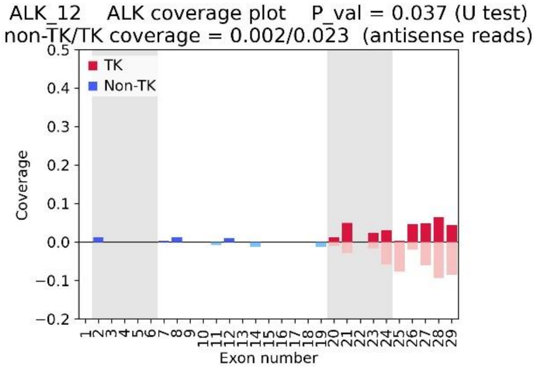
ALK_12 ALK coverage plot P_val = 0.037 (U test)
non-TK/TK coverage = 0.002/0.023 (antisense reads)
TK
Non-TK
Coverage
Exon number

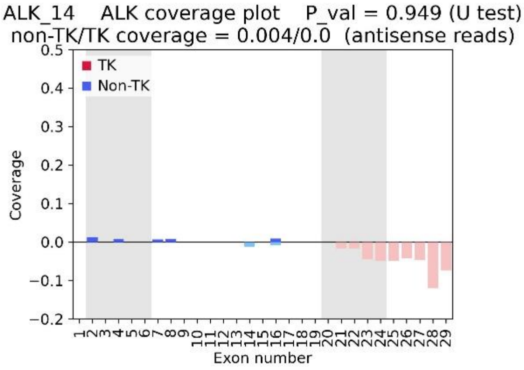
ALK_14 ALK coverage plot P_val = 0.949 (U test)
non-TK/TK coverage = 0.004/0.0 (antisense reads)
TK
Non-TK
Coverage
Exon number

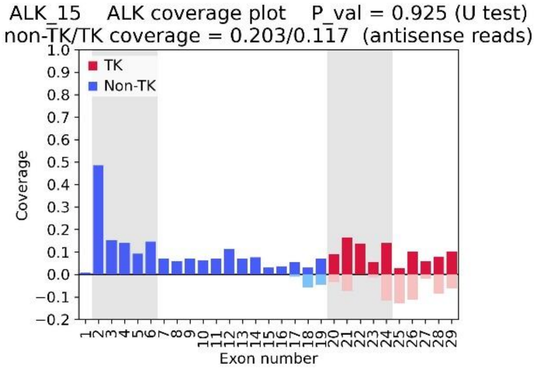
ALK_15 ALK coverage plot P_val = 0.925 (U test)
non-TK/TK coverage = 0.203/0.117 (antisense reads)
TK
Non-TK
Coverage
Exon number

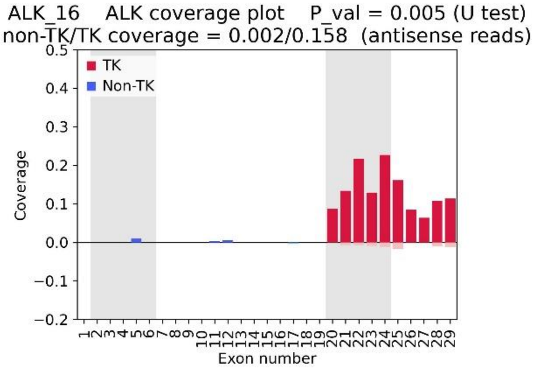
ALK_16 ALK coverage plot P_val = 0.005 (U test)
non-TK/TK coverage = 0.002/0.158 (antisense reads)
TK
Non-TK
Coverage
Exon number

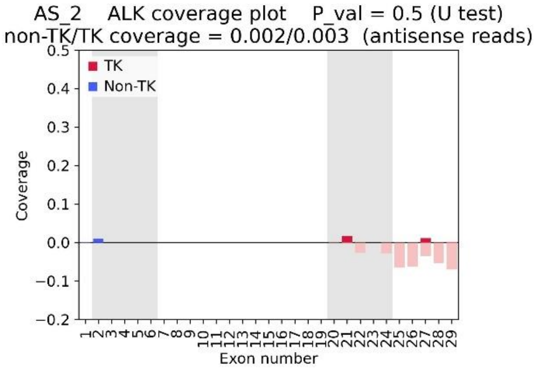
AS_2 ALK coverage plot P_val = 0.5 (U test)
non-TK/TK coverage = 0.002/0.003 (antisense reads)
TK
Non-TK
Coverage
Exon number

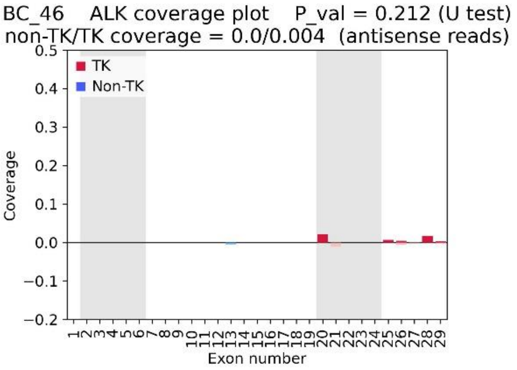
BC_46 ALK coverage plot P_val = 0.212 (U test)
non-TK/TK coverage = 0.0/0.004 (antisense reads)
TK
Non-TK
Coverage
Exon number

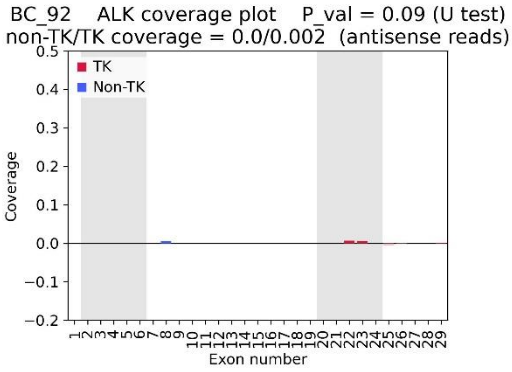
BC_92 ALK coverage plot P_val = 0.09 (U test)
non-TK/TK coverage = 0.0/0.002 (antisense reads)
TK
Non-TK
Coverage
Exon number

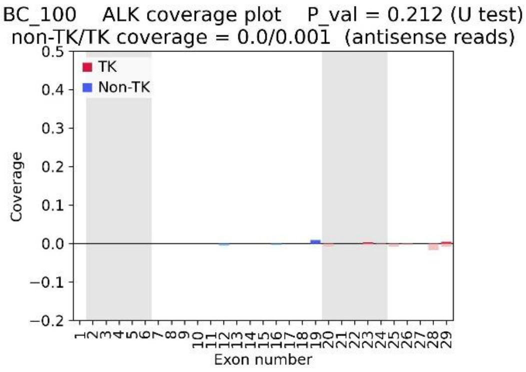
BC_100 ALK coverage plot P_val = 0.212 (U test)
non-TK/TK coverage = 0.0/0.001 (antisense reads)
TK
Non-TK
Coverage
Exon number

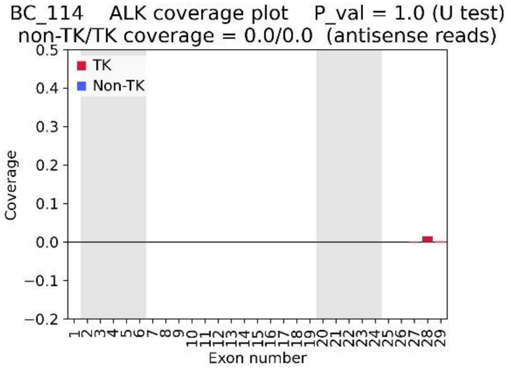
BC_114 ALK coverage plot P_val = 1.0 (U test)
non-TK/TK coverage = 0.0/0.0 (antisense reads)
TK
Non-TK
Coverage
Exon number

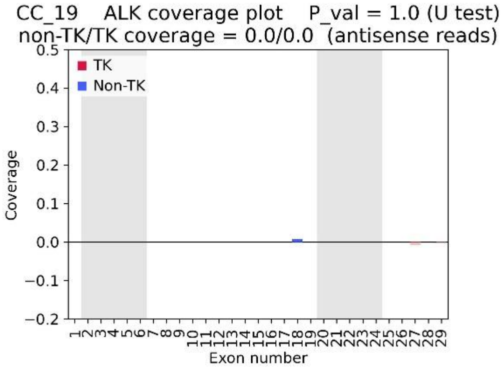
CC_19 ALK coverage plot P_val = 1.0 (U test)
non-TK/TK coverage = 0.0/0.0 (antisense reads)
TK
Non-TK
Coverage
Exon number

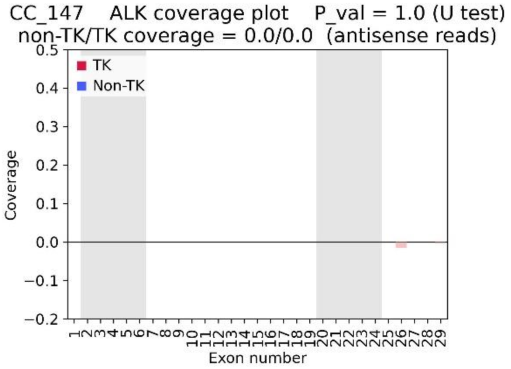
CC_147 ALK coverage plot P_val = 1.0 (U test)
non-TK/TK coverage = 0.0/0.0 (antisense reads)
TK
Non-TK
Coverage
Exon number

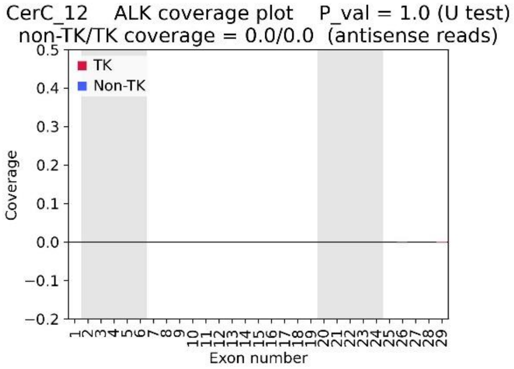
CerC_12 ALK coverage plot P_val = 1.0 (U test)
non-TK/TK coverage = 0.0/0.0 (antisense reads)
TK
Non-TK
Coverage
Exon number

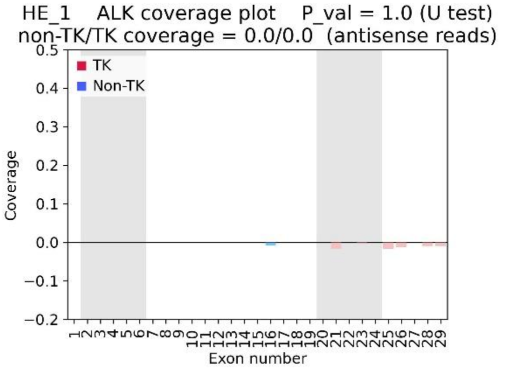
HE_1 ALK coverage plot P_val = 1.0 (U test)
non-TK/TK coverage = 0.0/0.0 (antisense reads)
TK
Non-TK
Coverage
Exon number

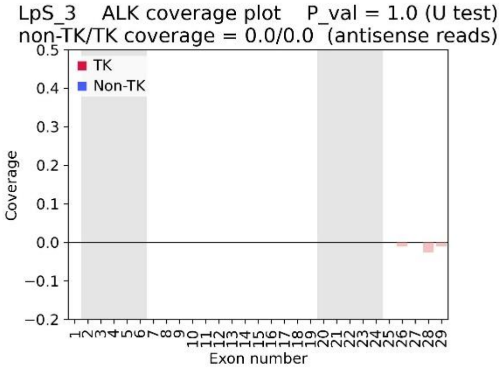
LpS_3 ALK coverage plot P_val = 1.0 (U test)
non-TK/TK coverage = 0.0/0.0 (antisense reads)
TK
Non-TK
Coverage
Exon number

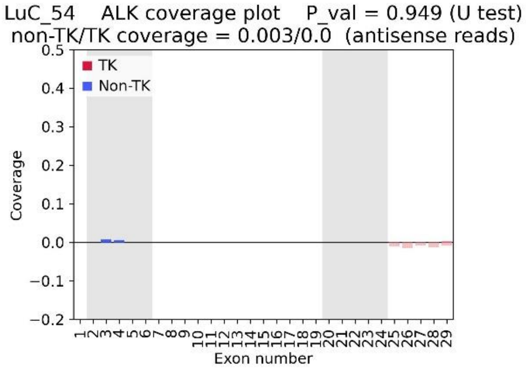
LuC_54 ALK coverage plot P_val = 0.949 (U test)
non-TK/TK coverage = 0.003/0.0 (antisense reads)
TK
Non-TK
Coverage
Exon number

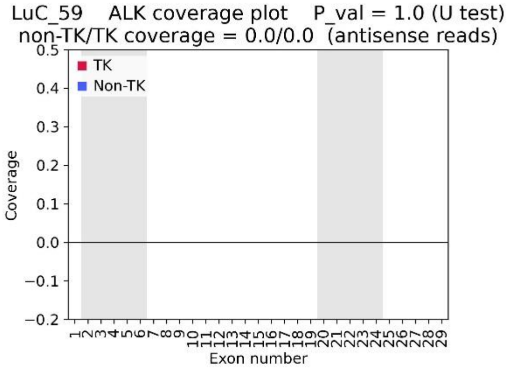
LuC_59 ALK coverage plot P_val = 1.0 (U test)
non-TK/TK coverage = 0.0/0.0 (antisense reads)
TK
Non-TK
Coverage
Exon number

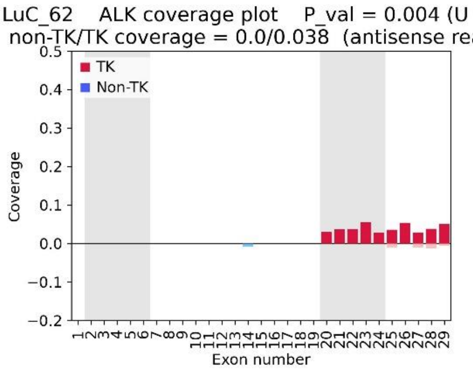
LuC_62 ALK coverage plot P_val = 0.004 (U test)
non-TK/TK coverage = 0.0/0.038 (antisense reads)
TK
Non-TK
Coverage
Exon number

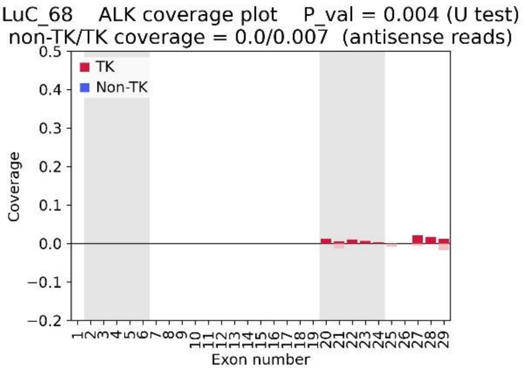
LuC_68 ALK coverage plot P_val = 0.004 (U test)
non-TK/TK coverage = 0.0/0.007 (antisense reads)
TK
Non-TK
Coverage
Exon number

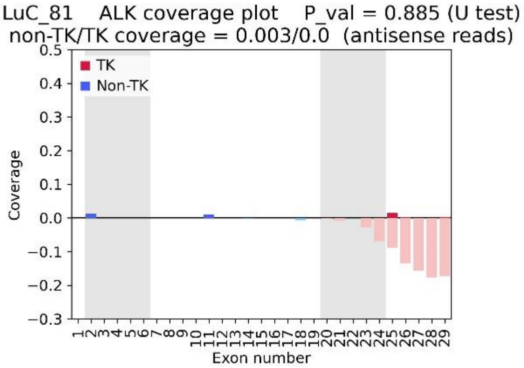
LuC_81 ALK coverage plot P_val = 0.885 (U test)
non-TK/TK coverage = 0.003/0.0 (antisense reads)
TK
Non-TK
Coverage
Exon number

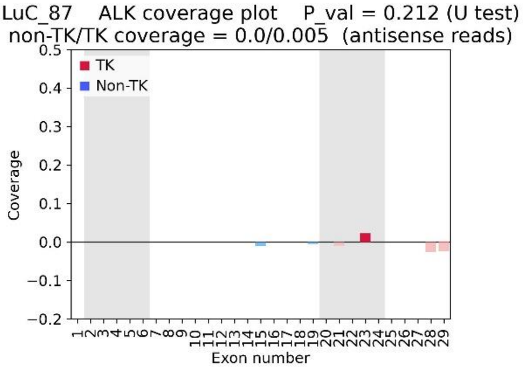
LuC_87 ALK coverage plot P_val = 0.212 (U test)
non-TK/TK coverage = 0.0/0.005 (antisense reads)
TK
Non-TK
Coverage
Exon number

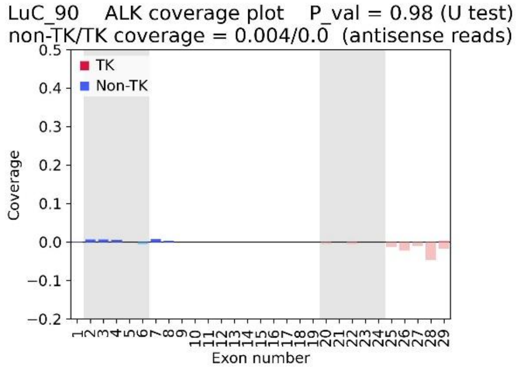
LuC_90 ALK coverage plot P_val = 0.98 (U test)
non-TK/TK coverage = 0.004/0.0 (antisense reads)
TK
Non-TK
Coverage
Exon number

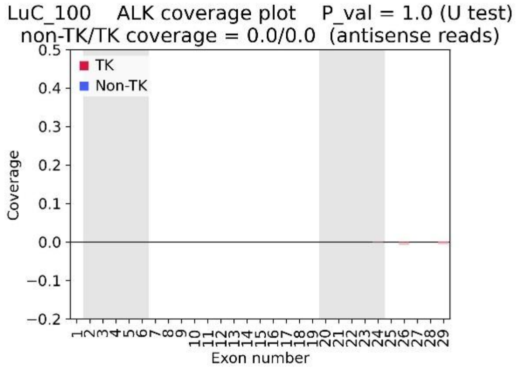
LuC_100 ALK coverage plot P_val = 1.0 (U test)
non-TK/TK coverage = 0.0/0.0 (antisense reads)
TK
Non-TK
Coverage
Exon number

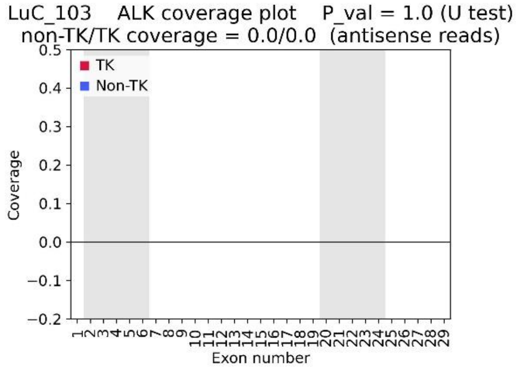
LuC_103 ALK coverage plot P_val = 1.0 (U test)
non-TK/TK coverage = 0.0/0.0 (antisense reads)
TK
Non-TK
Coverage
Exon number

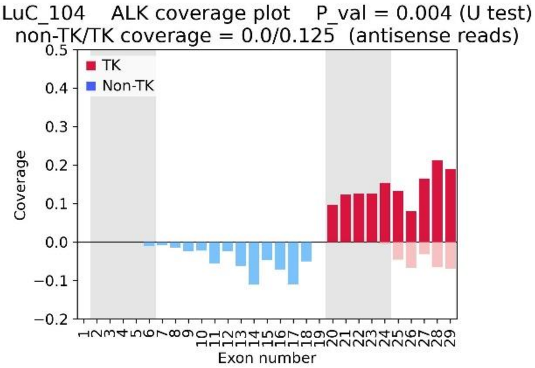
LuC_104 ALK coverage plot P_val = 0.004 (U test)
non-TK/TK coverage = 0.0/0.125 (antisense reads)
TK
Non-TK
Coverage
Exon number

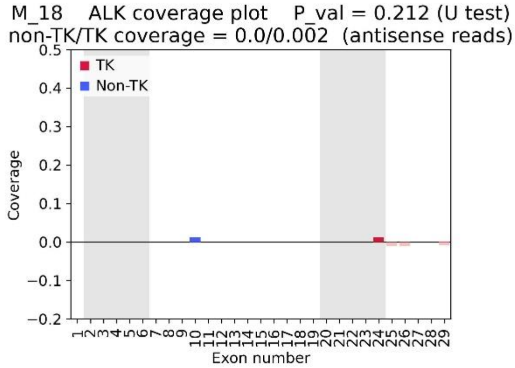
M_18 ALK coverage plot P_val = 0.212 (U test)
non-TK/TK coverage = 0.0/0.002 (antisense reads)
TK
Non-TK
Coverage
Exon number

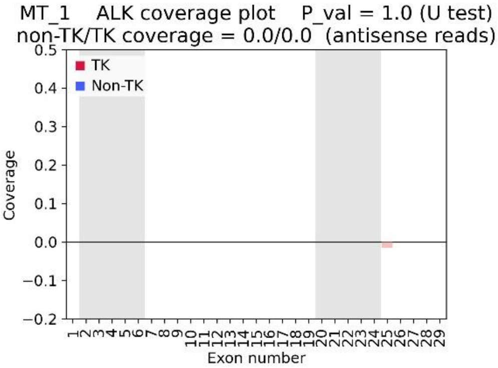
MT_1 ALK coverage plot P_val = 1.0 (U test)
non-TK/TK coverage = 0.0/0.0 (antisense reads)
TK
Non-TK
Coverage
Exon number

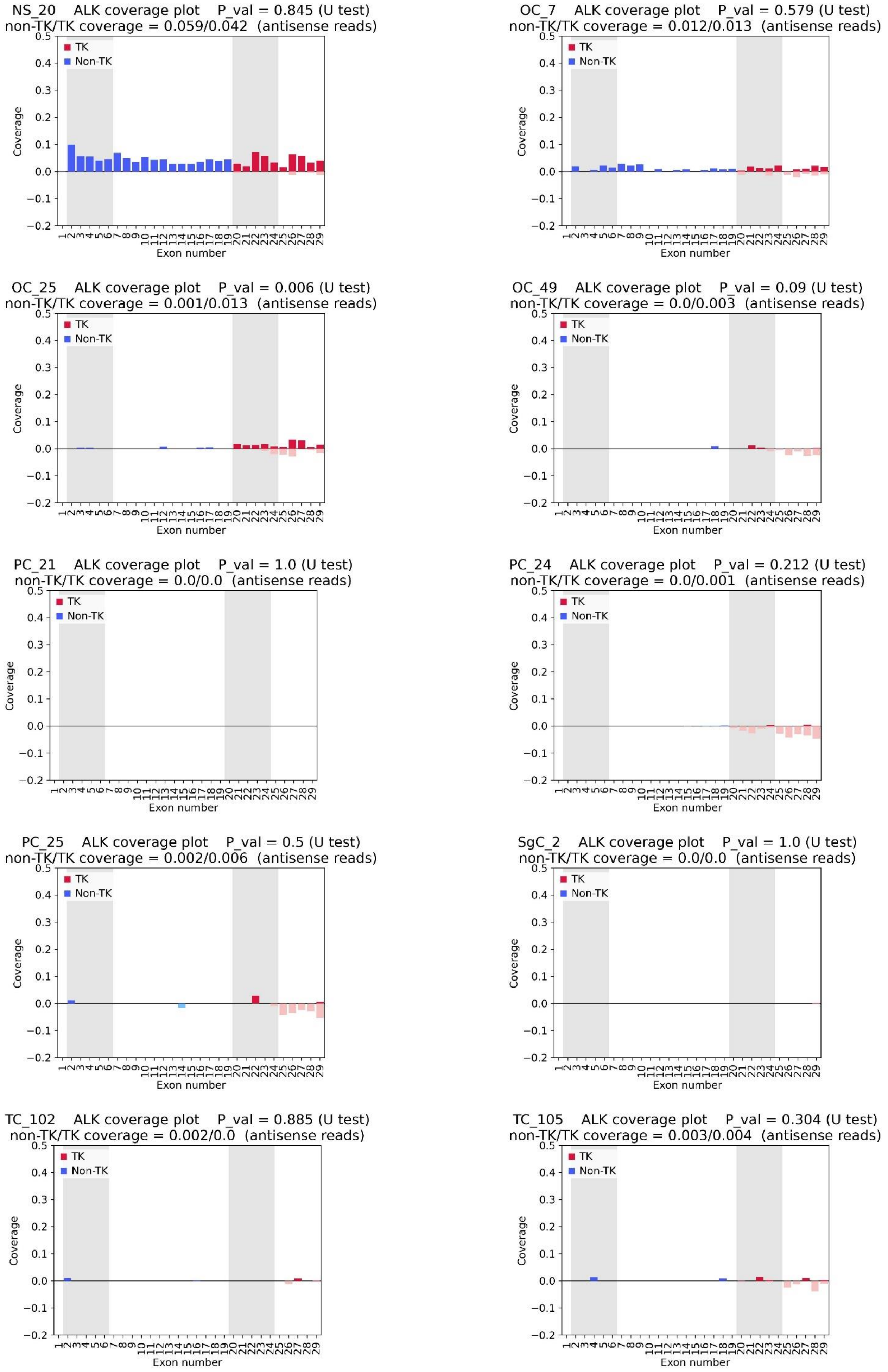

Figure S2. *ALK* coverage plots based on RNA-seq data, normalized on exon length and total read number in sample. Coverage of the *ALK*-sense reads is shown on the positive scale, while *ALK*- antisense reads are displayed on the negative scale. TK – tyrosine kinase domain-related exons; non-TK – exons not related to the tyrosine kinase domain; non-TK/TK coverage – ratio of mean coverage of five non-TK exons (exons 2-6) and five TK exons (exons 20-24).

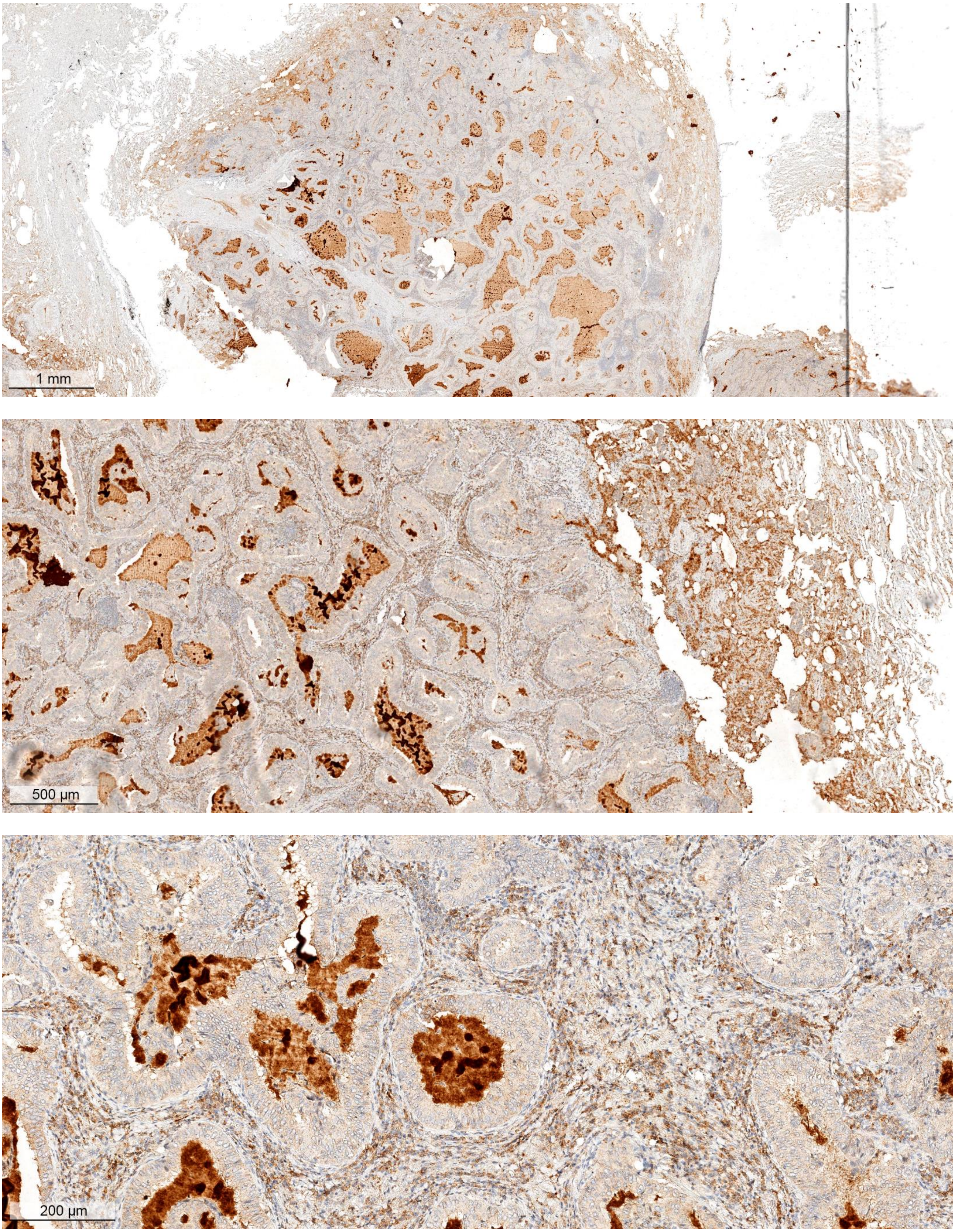

Figure S3. ALK immunostaining with clone D5F3 (Ventana) for sample LuC_103.

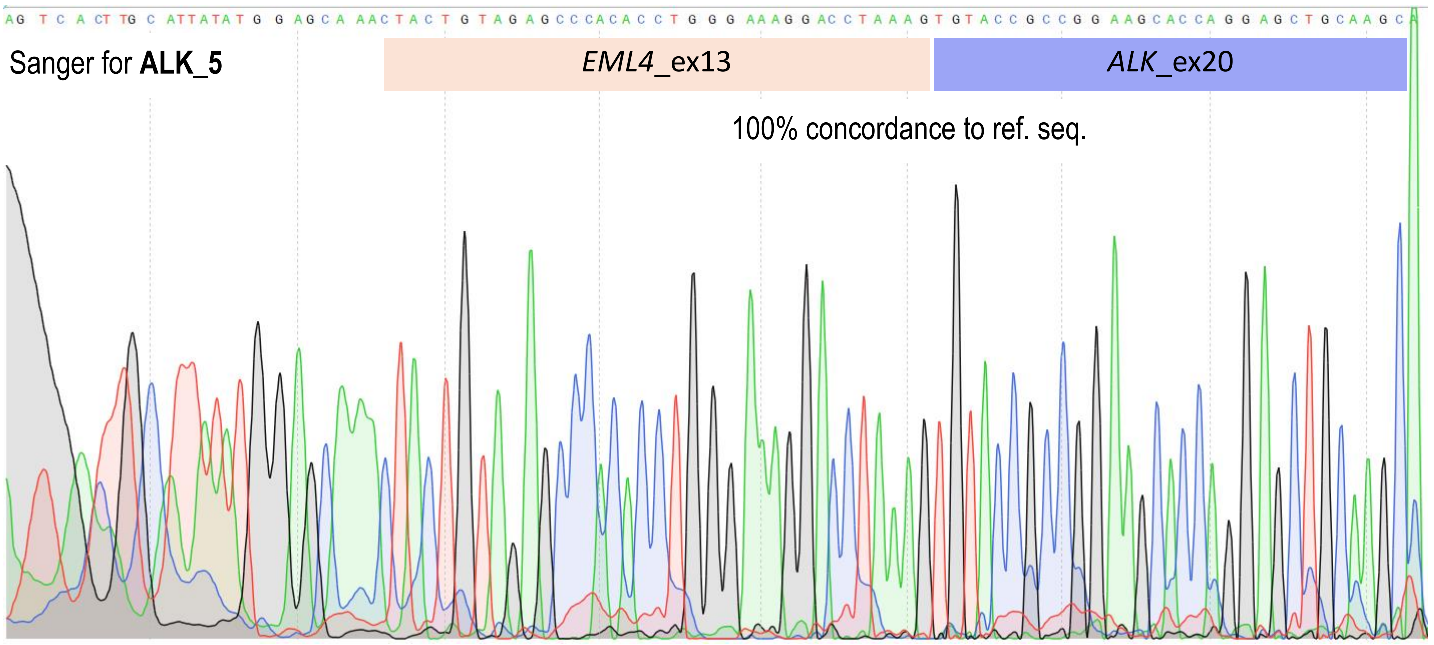
Sanger for **ALK_5**
*EML4*_ex13
*ALK*_ex20
100% concordance to ref. seq.

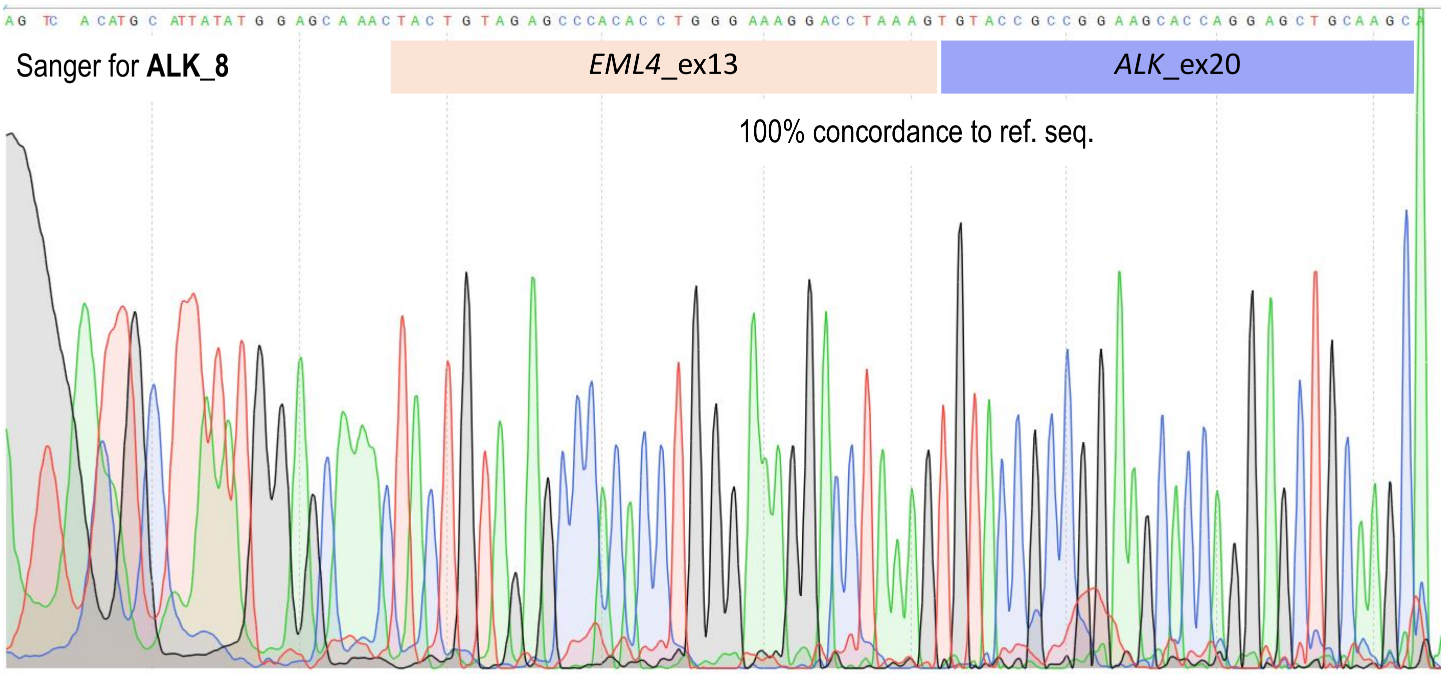
Sanger for **ALK_8**
*EML4*_ex13
*ALK*_ex20
100% concordance to ref. seq.

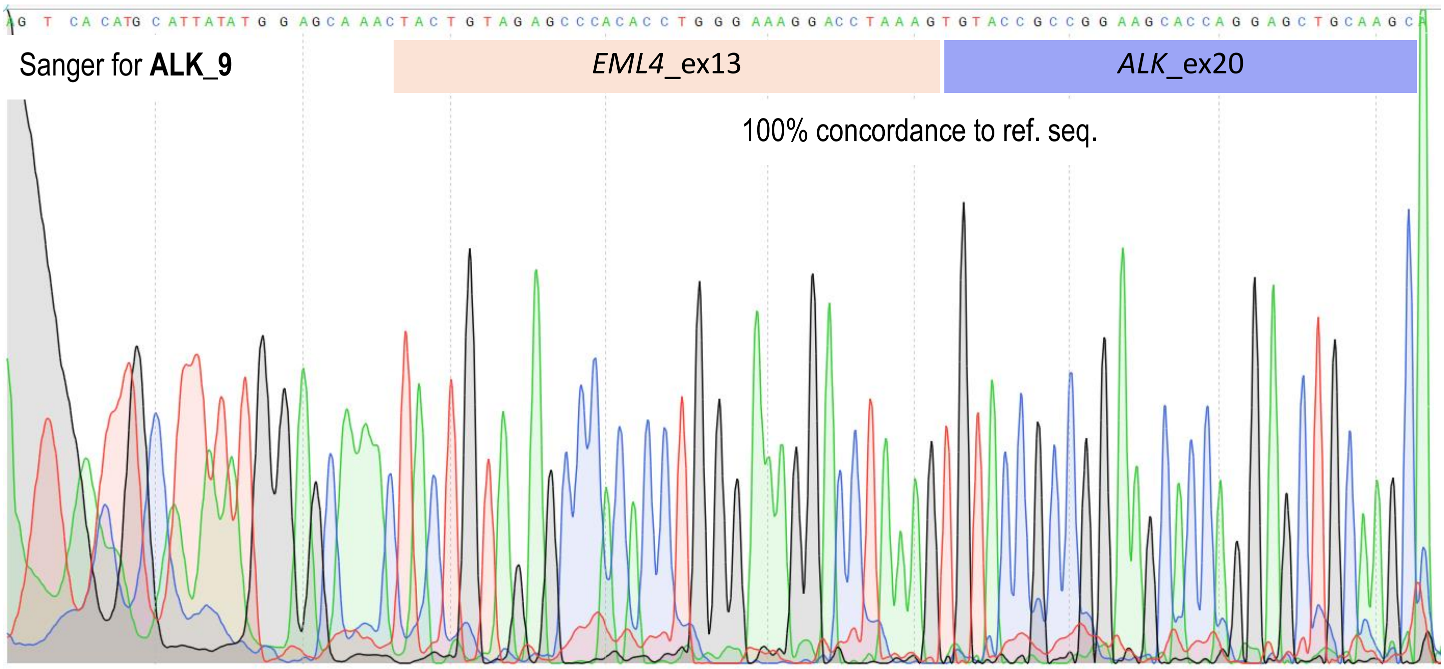
Sanger for **ALK_9**
*EML4*_ex13
*ALK*_ex20
100% concordance to ref. seq.

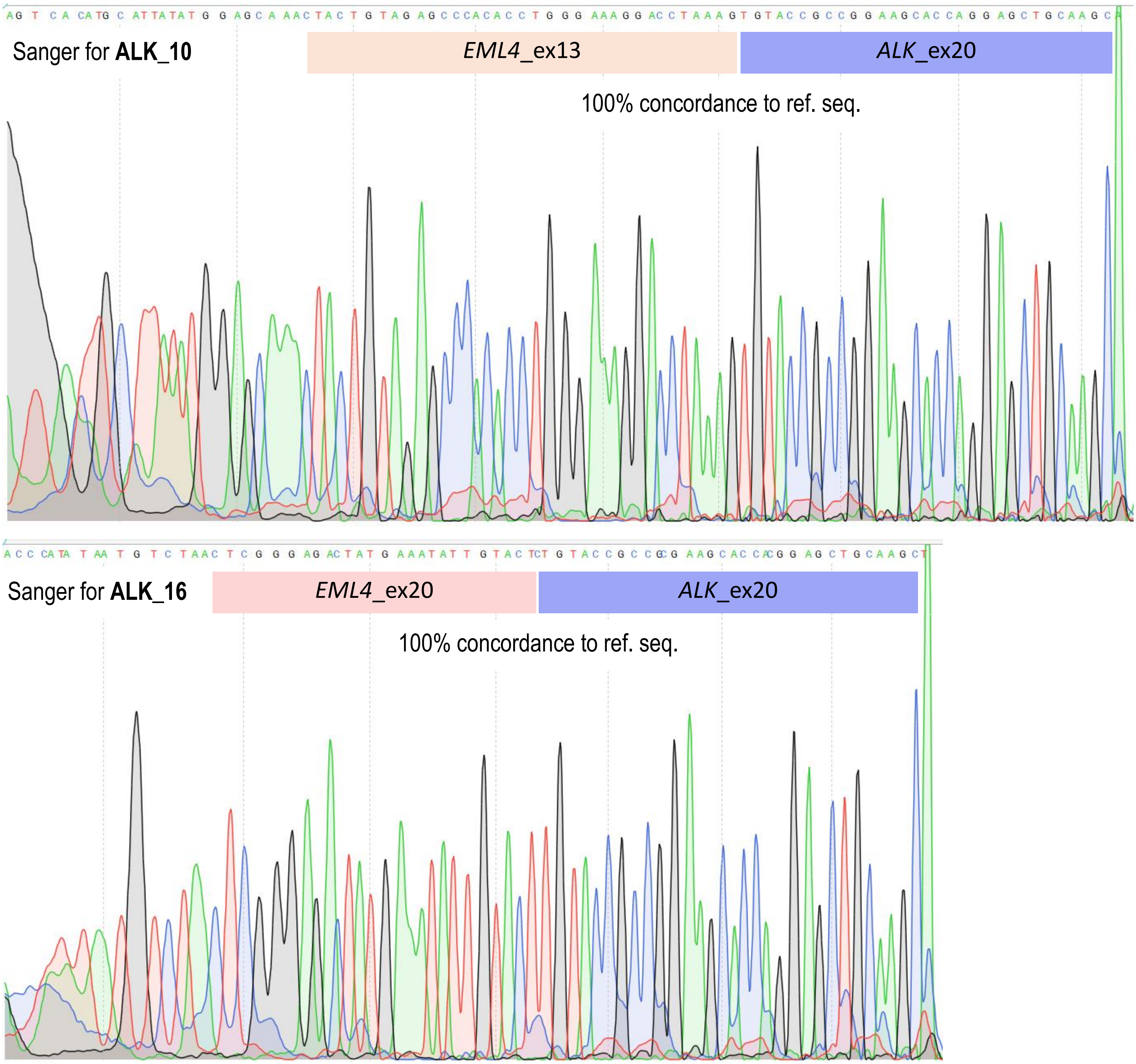

Figure S4. Results of *ALK* fusion validation by Sanger sequencing.

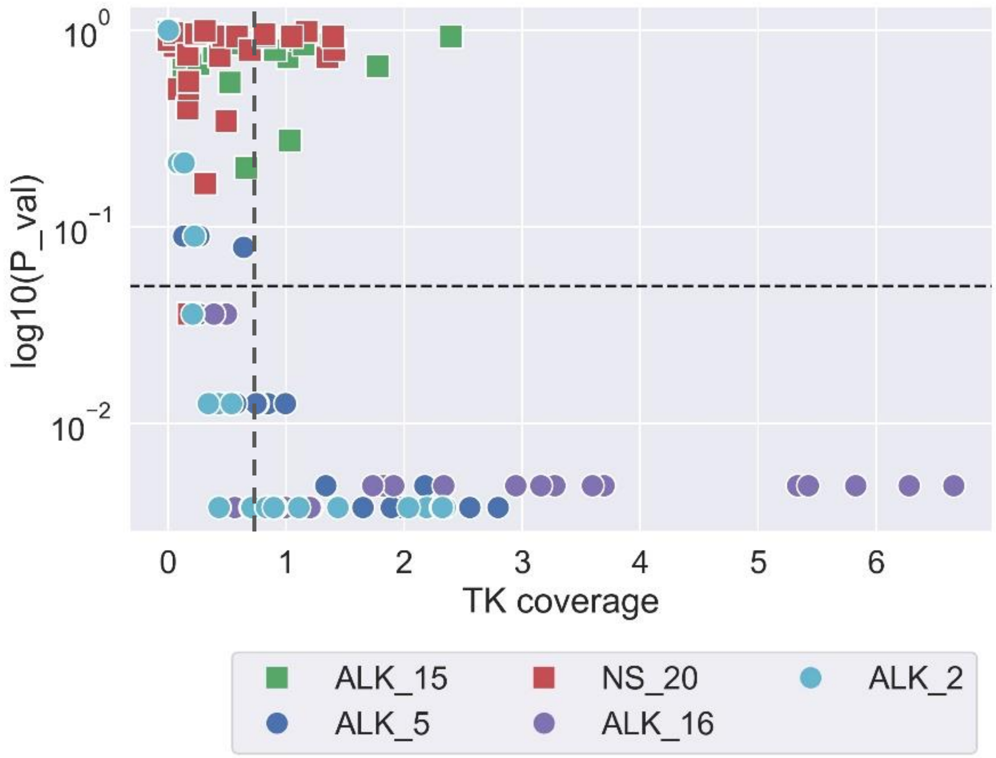

Figure S5. Dependence of statistical significance for *ALK* coverage asymmetry on the coverage depth of TK-related exons 20–24. Values are shown for subsamples of five samples, created by randomly selecting varying numbers of reads from the original FASTQ files. Round markers represent subsamples of true positive samples with *ALK* fusions, while square markers represent subsamples of true negative samples with wt-*ALK*. The horizontal dashed line denotes the threshold *p*-value of 0.05. The vertical dashed line indicates the simulated minimum coverage depth threshold, below which the detection of *ALK* coverage asymmetry in RNAseq is unreliable.

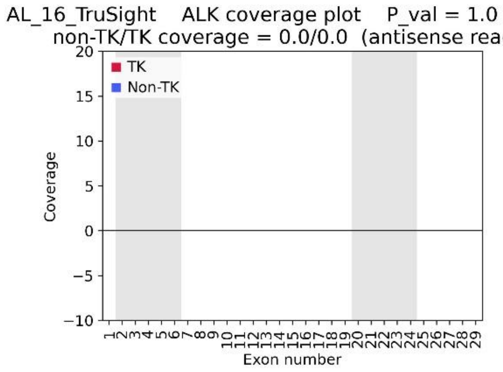
AL_16_TruSight ALK coverage plot P_val = 1.0 (U test)
non-TK/TK coverage = 0.0/0.0 (antisense reads)
TK
Non-TK
Coverage
Exon number

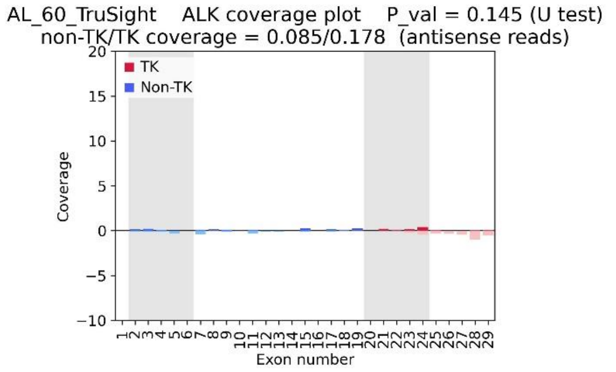
AL_60_TruSight ALK coverage plot P_val = 0.145 (U test)
non-TK/TK coverage = 0.085/0.178 (antisense reads)
TK
Non-TK
Coverage
Exon number

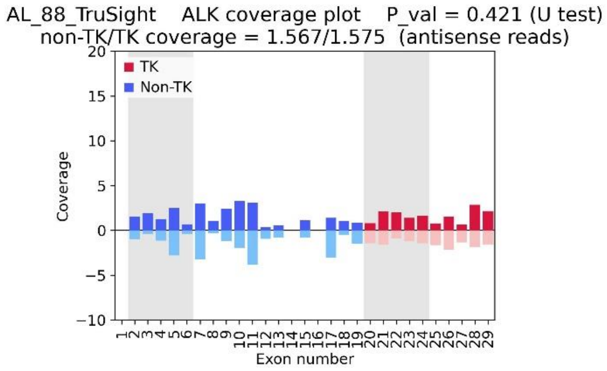
AL_88_TruSight ALK coverage plot P_val = 0.421 (U test)
non-TK/TK coverage = 1.567/1.575 (antisense reads)
TK
Non-TK
Coverage
Exon number

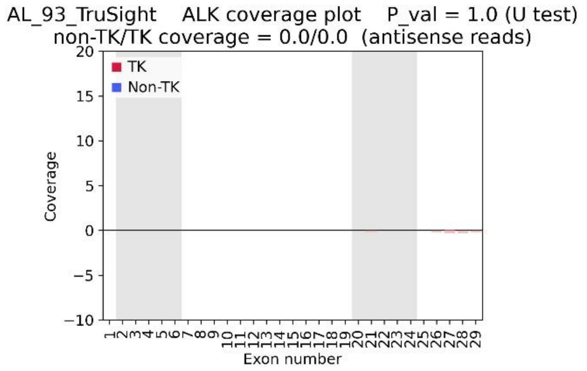
AL_93_TruSight ALK coverage plot P_val = 1.0 (U test)
non-TK/TK coverage = 0.0/0.0 (antisense reads)
TK
Non-TK
Coverage
Exon number

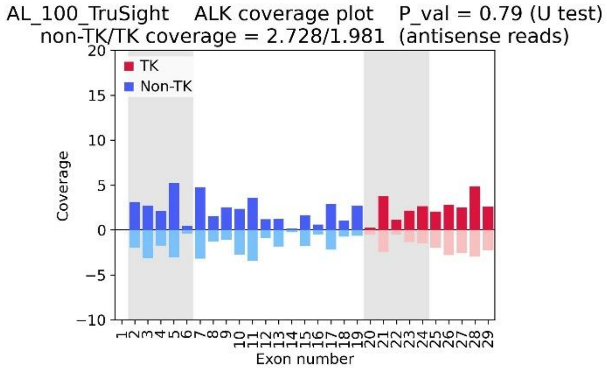
AL_100_TruSight ALK coverage plot P_val = 0.79 (U test)
non-TK/TK coverage = 2.728/1.981 (antisense reads)
TK
Non-TK
Coverage
Exon number

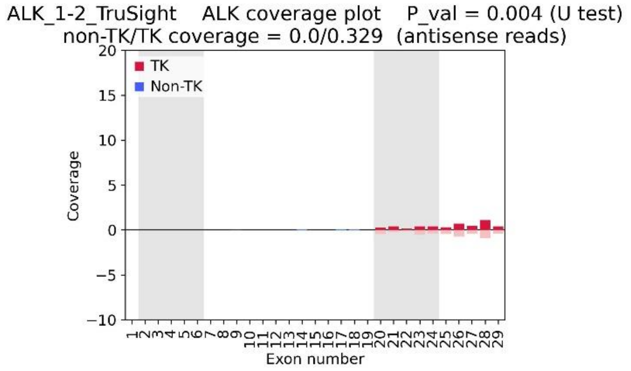
ALK_1-2_TruSight ALK coverage plot P_val = 0.004 (U test)
non-TK/TK coverage = 0.0/0.329 (antisense reads)
TK
Non-TK
Coverage
Exon number

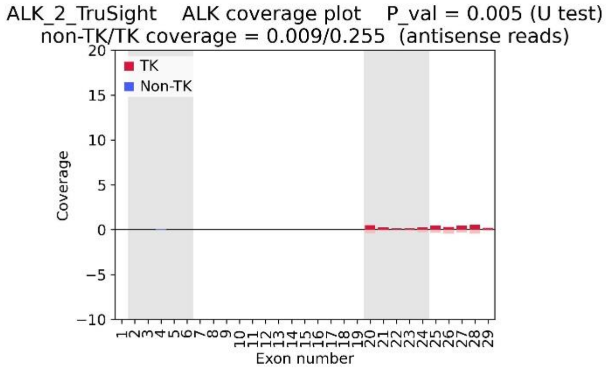
ALK_2_TruSight ALK coverage plot P_val = 0.005 (U test)
non-TK/TK coverage = 0.009/0.255 (antisense reads)
TK
Non-TK
Coverage
Exon number

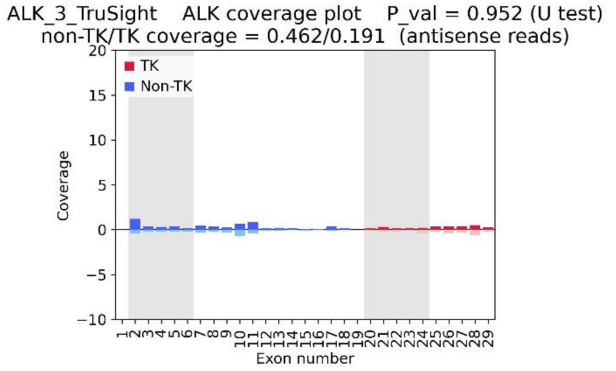
ALK_3_TruSight ALK coverage plot P_val = 0.952 (U test)
non-TK/TK coverage = 0.462/0.191 (antisense reads)
TK
Non-TK
Coverage
Exon number

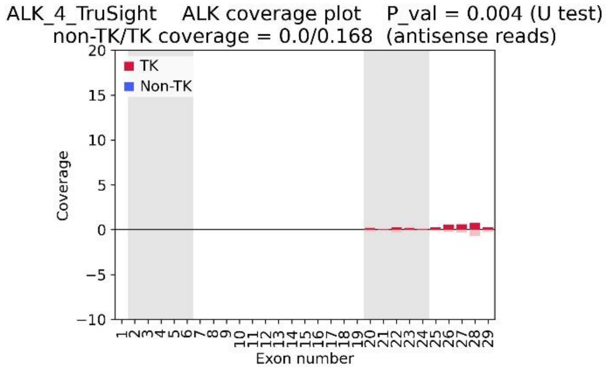
ALK_4_TruSight ALK coverage plot P_val = 0.004 (U test)
non-TK/TK coverage = 0.0/0.168 (antisense reads)
TK
Non-TK
Coverage
Exon number

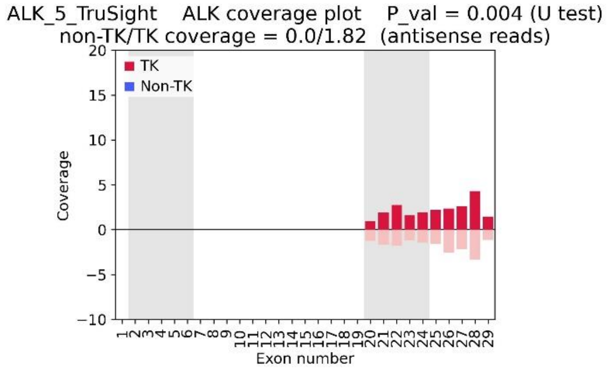
ALK_5_TruSight ALK coverage plot P_val = 0.004 (U test)
non-TK/TK coverage = 0.0/1.82 (antisense reads)
TK
Non-TK
Coverage
Exon number

ALK_6-2_TruSight ALK coverage plot P_val = 0.013 (U test)
non-TK/TK coverage = 0.0/0.202 (antisense reads)

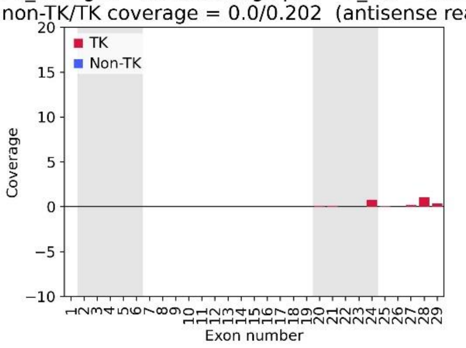

ALK_8_TruSight ALK coverage plot P_val = 0.006 (U test)
non-TK/TK coverage = 0.022/3.334 (antisense reads)

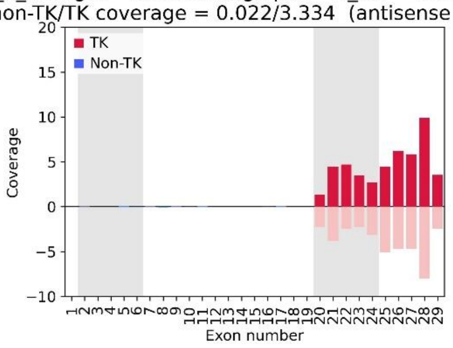

ALK_9_TruSight ALK coverage plot P_val = 0.004 (U test)
non-TK/TK coverage = 0.0/2.72 (antisense reads)

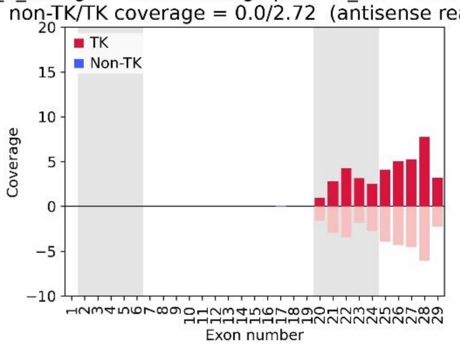

ALK_10_TruSight ALK coverage plot P_val = 0.006 (U test)
non-TK/TK coverage = 0.033/3.441 (antisense reads)

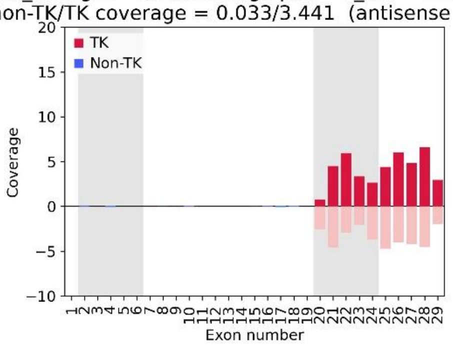

ALK_12_OncoFu_TruSight ALK coverage plot P_val = 0.004 (U test)
non-TK/TK coverage = 0.694/9.624 (antisense reads)

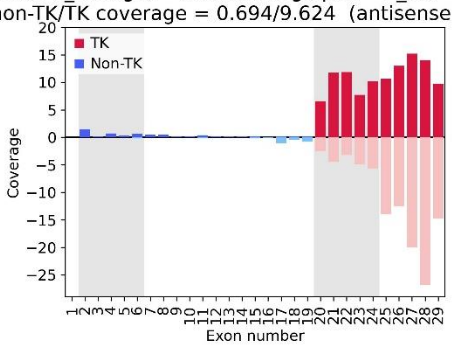

ALK_14_TruSight ALK coverage plot P_val = 0.017 (U test)
non-TK/TK coverage = 0.054/0.267 (antisense reads)

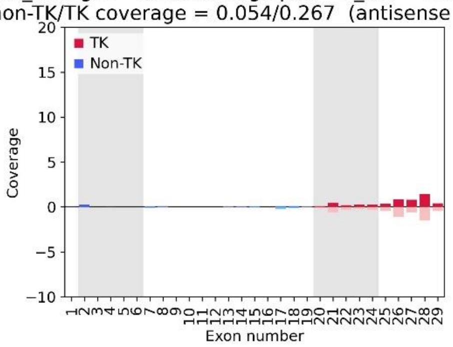

ALK_15_TruSight ALK coverage plot P_val = 0.889 (U test)
non-TK/TK coverage = 3.829/2.339 (antisense reads)

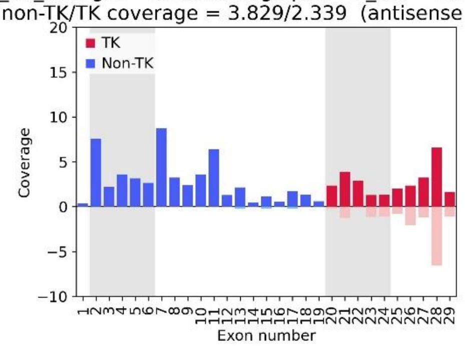

ALK_16_TruSight ALK coverage plot P_val = 0.004 (U test)
non-TK/TK coverage = 0.0/0.745 (antisense reads)

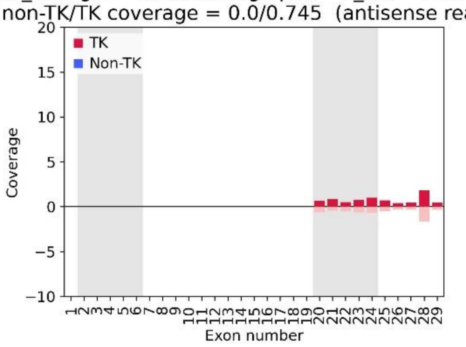

BC_46_TruSight ALK coverage plot P_val = 0.885 (U test)
non-TK/TK coverage = 0.019/0.0 (antisense reads)

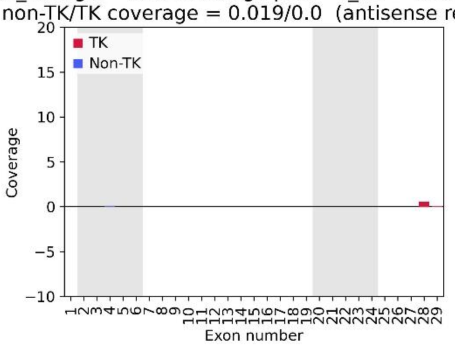

BC_92_TruSight ALK coverage plot P_val = 0.913 (U test)
non-TK/TK coverage = 0.334/0.138 (antisense reads)

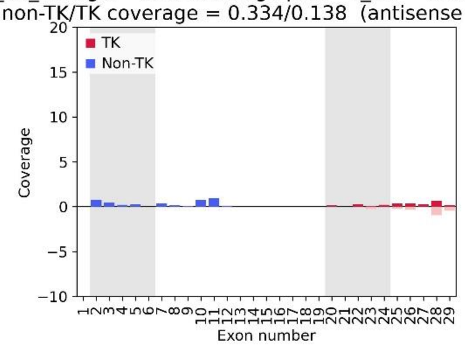

BC_100_TruSight ALK coverage plot P_val = 0.547 (U test)
non-TK/TK coverage = 0.023/0.033 (antisense reads)

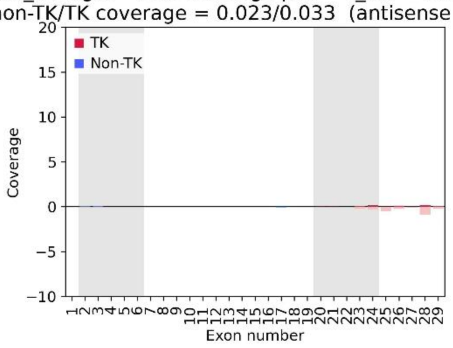

BC_114_TruSight ALK coverage plot P_val = 0.24 (U test)
non-TK/TK coverage = 0.032/0.065 (antisense reads)

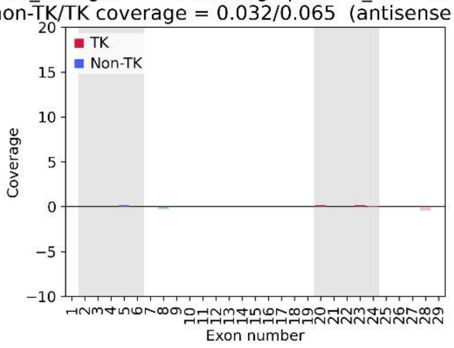

CC_19_TruSight ALK coverage plot P_val = 0.998 (U test)
non-TK/TK coverage = 0.205/0.0 (antisense reads)

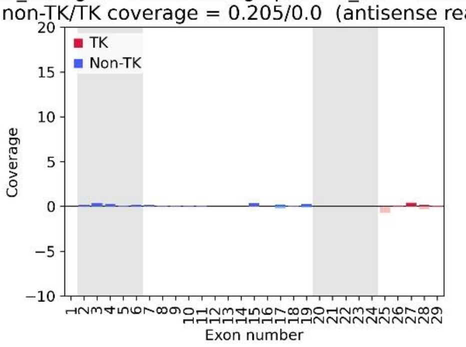

CC_147_TruSight ALK coverage plot P_val = 1.0 (U test)
non-TK/TK coverage = 0.0/0.0 (antisense reads)

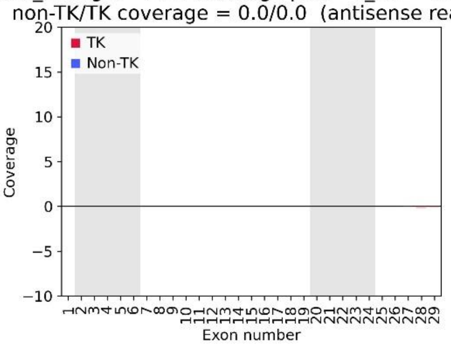

CerC_12_TruSight ALK coverage plot P_val = 0.09 (U test)
non-TK/TK coverage = 0.0/0.052 (antisense reads)

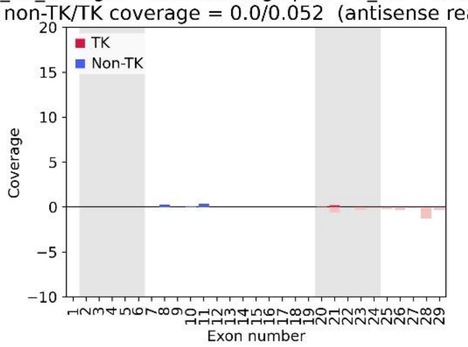

LpS_3_1_TruSight ALK coverage plot P_val = 0.12 (U test)
non-TK/TK coverage = 0.012/0.091 (antisense reads)

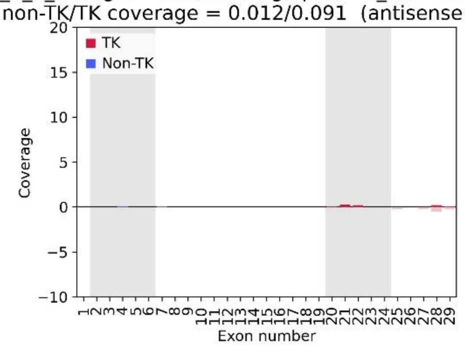

LuC_54_TruSight ALK coverage plot P_val = 0.664 (U test)
non-TK/TK coverage = 0.196/0.124 (antisense reads)

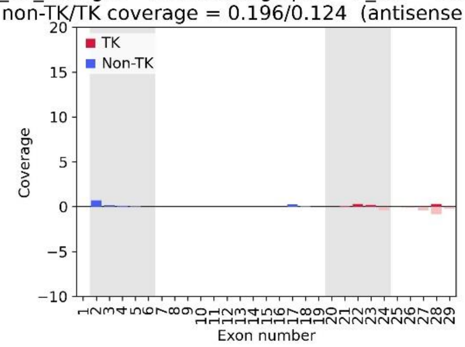

LuC_62_TruSight ALK coverage plot P_val = 0.006 (U test)
non-TK/TK coverage = 0.138/2.029 (antisense reads)

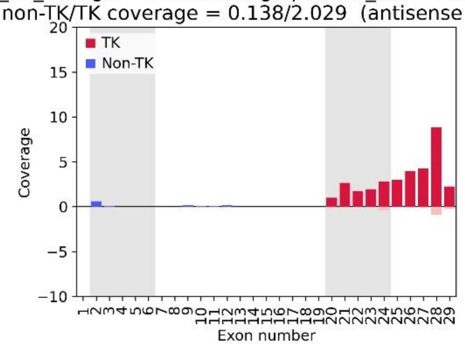

LuC_68_TruSight ALK coverage plot P_val = 0.004 (U test)
non-TK/TK coverage = 0.0/0.064 (antisense reads)

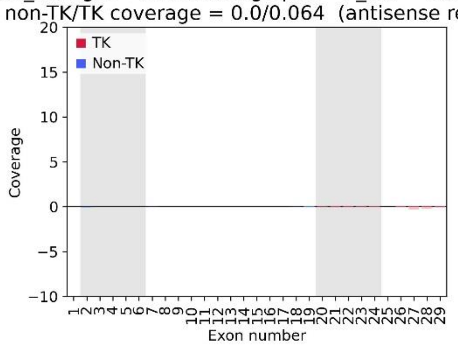

LuC_81_TruSight ALK coverage plot P_val = 0.263 (U test)
non-TK/TK coverage = 0.092/0.131 (antisense reads)

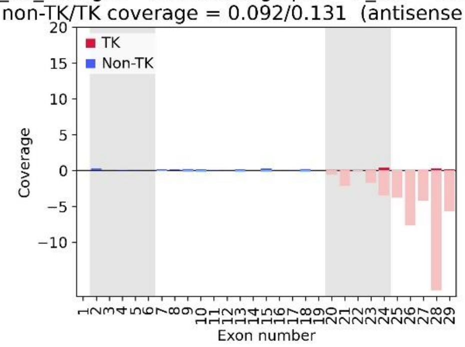

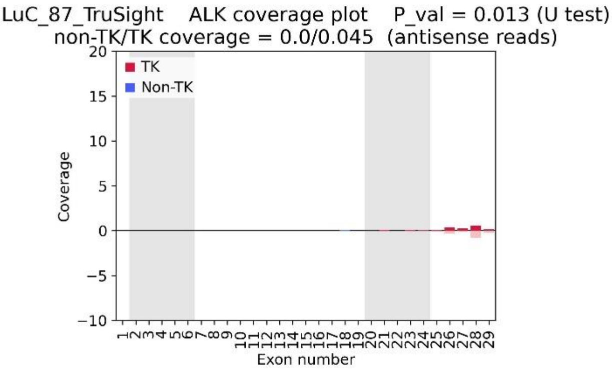
LuC_87_TruSight ALK coverage plot P_val = 0.013 (U test)
non-TK/TK coverage = 0.0/0.045 (antisense reads)
TK
Non-TK
Coverage
Exon number

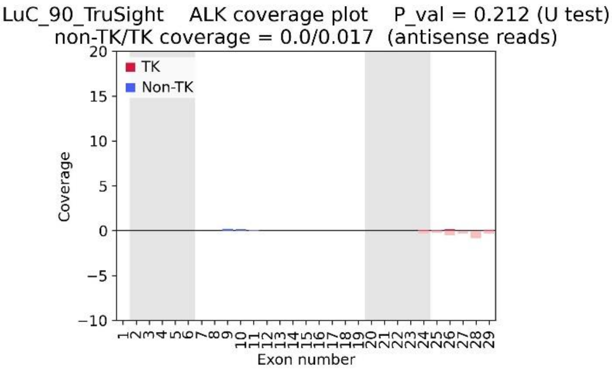
LuC_90_TruSight ALK coverage plot P_val = 0.212 (U test)
non-TK/TK coverage = 0.0/0.017 (antisense reads)
TK
Non-TK
Coverage
Exon number

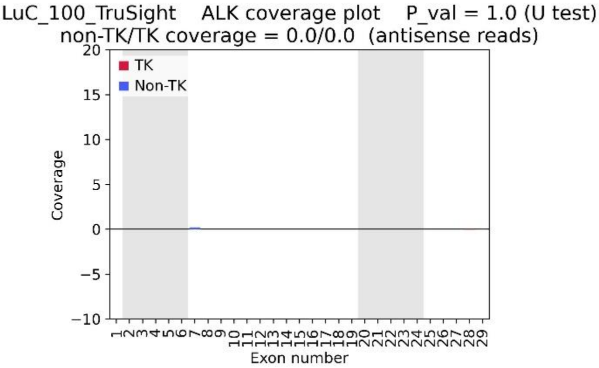
LuC_100_TruSight ALK coverage plot P_val = 1.0 (U test)
non-TK/TK coverage = 0.0/0.0 (antisense reads)
TK
Non-TK
Coverage
Exon number

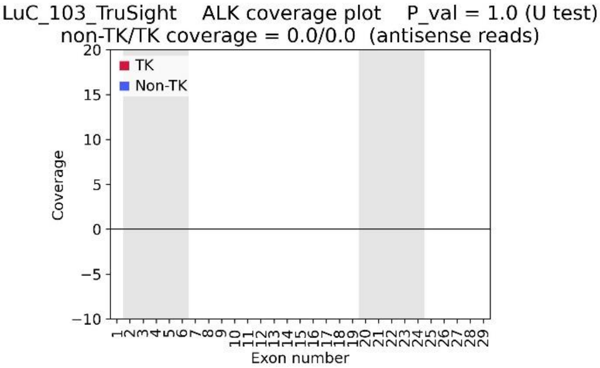
LuC_103_TruSight ALK coverage plot P_val = 1.0 (U test)
non-TK/TK coverage = 0.0/0.0 (antisense reads)
TK
Non-TK
Coverage
Exon number

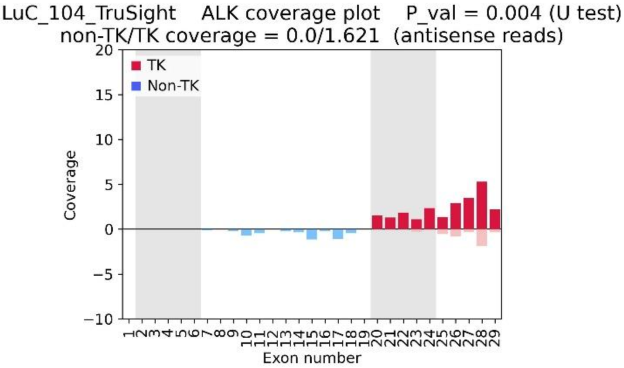
LuC_104_TruSight ALK coverage plot P_val = 0.004 (U test)
non-TK/TK coverage = 0.0/1.621 (antisense reads)
TK
Non-TK
Coverage
Exon number

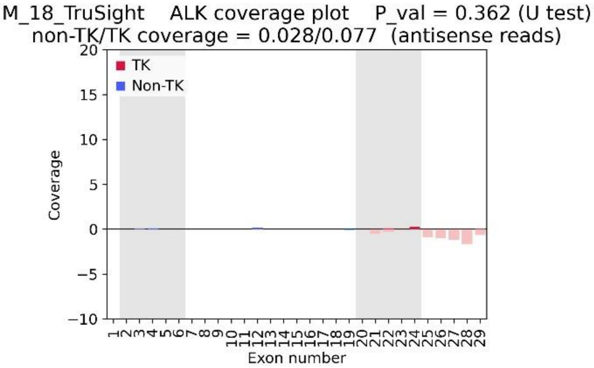
M_18_TruSight ALK coverage plot P_val = 0.362 (U test)
non-TK/TK coverage = 0.028/0.077 (antisense reads)
TK
Non-TK
Coverage
Exon number

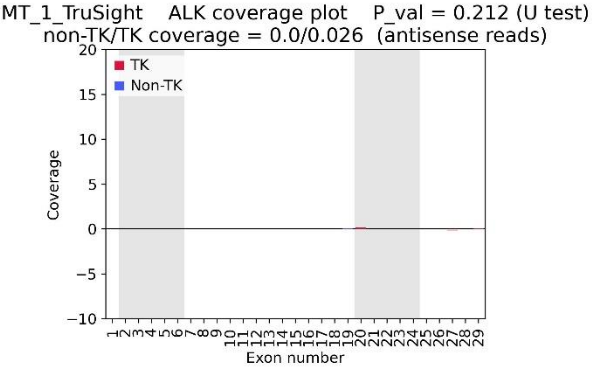
MT_1_TruSight ALK coverage plot P_val = 0.212 (U test)
non-TK/TK coverage = 0.0/0.026 (antisense reads)
TK
Non-TK
Coverage
Exon number

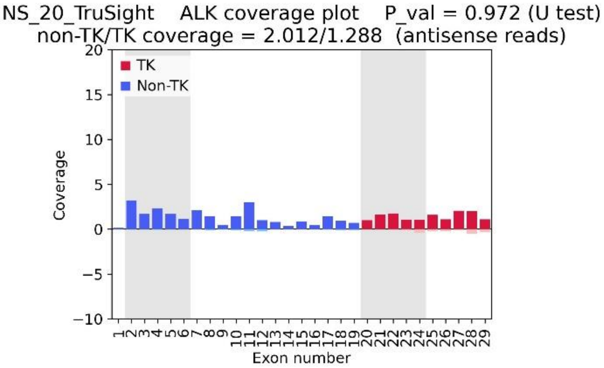
NS_20_TruSight ALK coverage plot P_val = 0.972 (U test)
non-TK/TK coverage = 2.012/1.288 (antisense reads)
TK
Non-TK
Coverage
Exon number

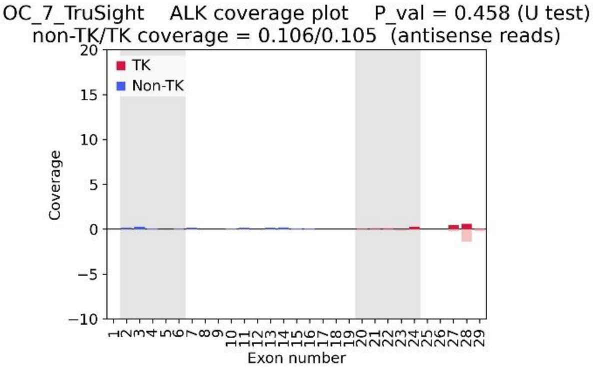
OC_7_TruSight ALK coverage plot P_val = 0.458 (U test)
non-TK/TK coverage = 0.106/0.105 (antisense reads)
TK
Non-TK
Coverage
Exon number

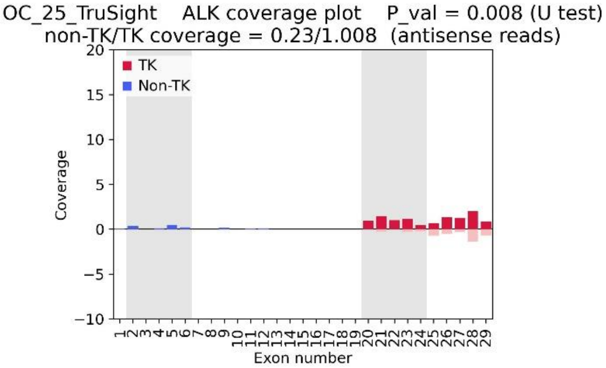
OC_25_TruSight ALK coverage plot P_val = 0.008 (U test)
non-TK/TK coverage = 0.23/1.008 (antisense reads)
TK
Non-TK
Coverage
Exon number

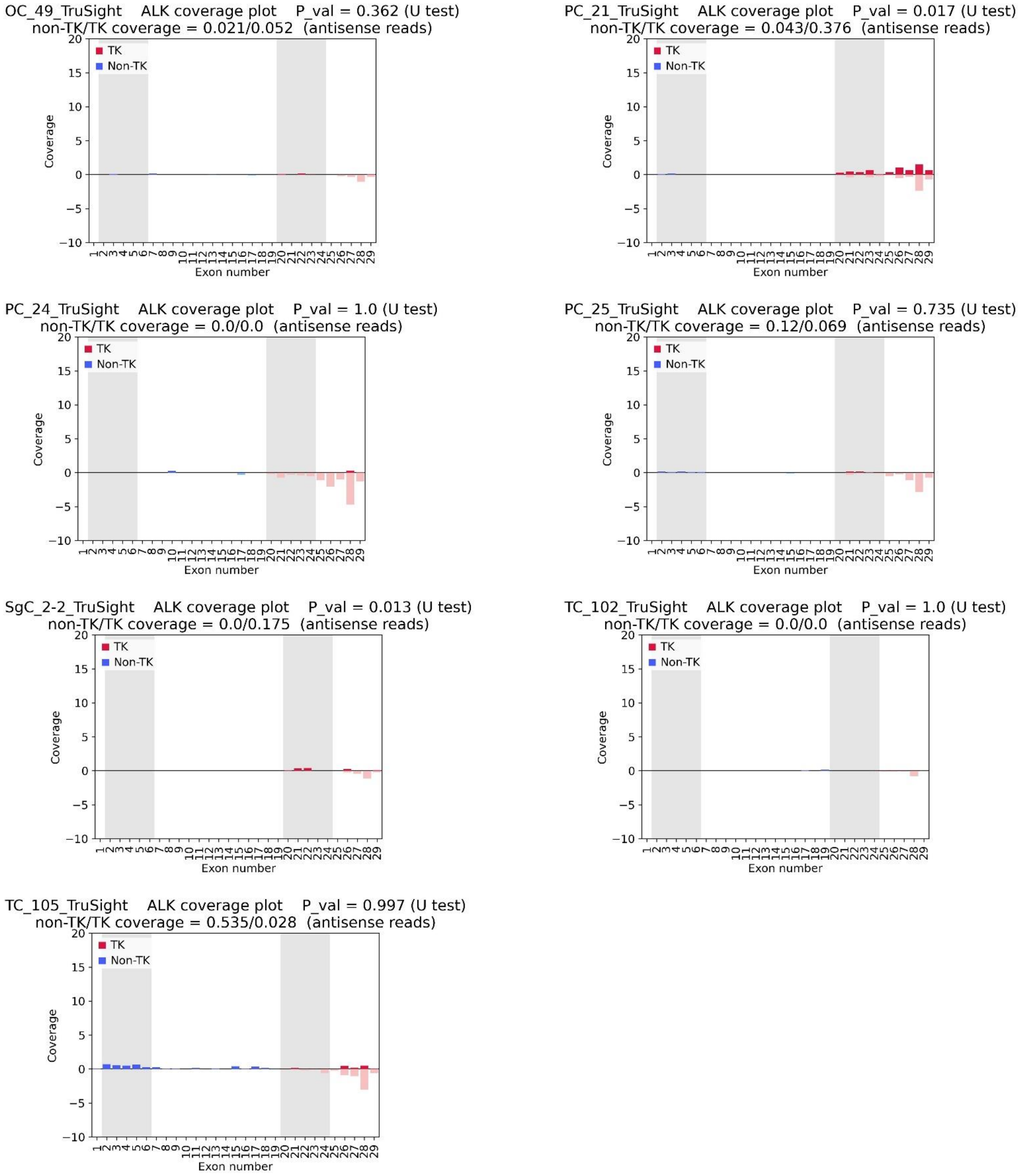

Figure S6. *ALK* coverage plots based on targeted NGS data (TruSight panel), normalized on exon length and total read number in sample. Coverage of the *ALK*-sense reads is shown on the positive scale, while *ALK*- antisense reads are displayed on the negative scale. TK – tyrosine kinase domain-related exons; non-TK – exons not related to the tyrosine kinase domain; non-TK/TK coverage – ratio of mean coverage of five non-TK exons (exons 2-6) and five TK exons (exons 20-24).
